# AVN944 Elicits Apoptotic Responses and Impedes Tumorigenic Potential in Ewing's Sarcoma Cells

**DOI:** 10.7150/ijbs.116651

**Published:** 2026-01-01

**Authors:** Hanah Lim, Seonock Lee, Gamin Kim, Eun Joo Lee, Jungho Kim

**Affiliations:** 1 Laboratory of Molecular and Cellular Biology, Department of Life Science, Sogang University, Seoul 04107, Korea.; 2 Stress-Responding Bionanomaterial Center, Sogang University, Seoul 04107, Korea.

**Keywords:** Ewing' sarcoma, AVN944, IMPDH2, Apoptosis, Tumorigenicity.

## Abstract

Inosine monophosphate dehydrogenase 2 (IMPDH2) is implicated in survival and proliferation of cancer cells because of its role in guanine nucleotide biosynthesis. This study evaluates the efficacy of AVN944, an IMPDH2 inhibitor, as a treatment for Ewing's sarcoma, a challenging malignancy in pediatric and young adult patients. Gene expression data, as well as clinical outcomes for sarcoma patients, from The Cancer Genome Atlas (TCGA) were analyzed to determine the association between IMPDH2 expression and survival. Human Ewing's sarcoma cell lines and xenograft models were used to evaluate the cellular and *in vivo* effects, respectively, of AVN944. Various cellular assays, including western blotting, MTT, BrdU incorporation, and colony formation assays, were conducted to assess the impact of AVN944 on proliferation, viability, and apoptosis. IC_50_ values were calculated from dose-response curves. Sarcoma patients with high expression of IMPDH2 showed a trend towards poorer overall survival. *In vitro*, AVN944 decreased the viability and proliferation of TC71 and SK-ES-1 Ewing's sarcoma cell lines significantly, and in a dose-dependent manner. The drug induced G_1_ cell cycle arrest and apoptosis, as evidenced by increased expression of pro-apoptotic markers and reduced expression of cell cycle proteins. *In vivo*, AVN944 effectively inhibited tumor growth in xenograft models without notable toxicity. The IC_50_ of AVN944 was approximately 0.05 μM for both TC71 and SK-ES-1 cell lines. Thus, AVN944 displays potent anti-tumor activity against Ewing's sarcoma cells both *in vitro* and *in vivo* by inhibiting IMPDH2. The inhibitor causes cell cycle arrest and apoptosis, significantly reducing tumor viability and proliferation. These findings highlight the therapeutic potential of targeting nucleotide biosynthesis pathways in Ewing's sarcoma, suggesting that AVN944 could be a valuable addition to existing treatment protocols. Further clinical investigations are recommended to validate these preclinical outcomes and to explore integration of AVN944 into treatment regimens for Ewing's sarcoma.

## Introduction

Sarcomas are a complex and heterogeneous group of cancers derived from mesenchymal progenitor cells of bone, muscle, and connective tissue 1, 2. These malignancies are broadly categorized as either bone sarcomas or soft tissue sarcomas, each encompassing numerous subtypes that differ significantly with respect to clinical behavior, treatment response, and prognosis 3. Bone sarcomas, including osteosarcoma, chondrosarcoma, and Ewing's sarcoma, typically affect the pediatric population, and are notable for their dismal prognosis and substantial impact on morbidity 4. Soft tissue sarcomas, which are more prevalent in adults, can develop from fat, muscle, nerve, fibrous tissues, and blood vessels, and have the potential to metastasize at a late stage 5.

Ewing's sarcoma is a highly malignant form of bone sarcoma occurring most commonly in adolescents and young adults 6, 7. The disease accounts for a significant percentage of bone cancer cases in children, and is characterized by a distinctive genetic mutation, most notably the EWS-Fli1 fusion gene 8, 9. It primarily affects the long bones, pelvis, and chest wall, but can also appear at unusual sites such as the skull or spinal bones. The clinical presentation of Ewing's sarcoma often includes intense pain, swelling at the tumor site, and systemic symptoms such as fever or weight loss, which can be mistaken for other adolescent conditions and lead to delays in diagnosis 7, 10. The aggressive nature of Ewing's sarcoma, and its propensity for early metastasis to the lungs and other bones, necessitate advancements in therapeutic strategies 6, 7, 11. The challenge is not only to improve survival rates but also to mitigate the long-term effects of current high-dose chemotherapeutic regimes, which are particularly burdensome for young patients.

Inosine monophosphate dehydrogenase 2 (IMPDH2), a pivotal enzyme in the purine nucleotide biosynthesis pathway, is involved specifically in the synthesis of xanthosine monophosphate (XMP) from inosine monophosphate (IMP). Its enzymatic activity is crucial for production of guanine nucleotides, which are essential components of both RNA and DNA 12, 13. The proliferative demands of cancer cells necessitate an increased supply of these nucleotides, underlining the critical role of IMPDH2 in cell growth and replication. A growing body of research demonstrates a correlation between elevated expression of IMPDH2 and the aggressiveness of various cancers, including leukemia, lymphoma, and several solid tumors 14-16. Elevated IMPDH2 activity supports a heightened proliferative rate and increased survival capabilities of cancer cells, thereby contributing to tumor progression and poor clinical outcomes. This association has been noted particularly in studies showing that cancer cells often upregulate IMPDH2 as a compensatory mechanism to meet their increased metabolic demands, highlighting its potential as a therapeutic target 12-16. These findings provide a compelling rationale for exploration of IMPDH2 inhibitors such as AVN944 in the context of cancer treatment, setting the stage for their evaluation in clinical trials aimed at improving outcomes for patients with malignancies characterized by high IMPDH2 activity.

AVN944 (VX-944, Vertex Pharmaceuticals, Cambridge, MA, USA) is an IMPDH2 inhibitor designed to disrupt the metabolic pathways crucial for tumor cell proliferation and survival. As an enzyme essential for the guanine nucleotide biosynthesis pathway, IMPDH2 plays a critical role in the metabolic processes that fuel cell division and growth, particularly in cancer cells 16, 17. Inhibiting this pathway can have significant therapeutic benefits, especially in cancers in which nucleotide synthesis is upregulated. AVN944 stands out among IMPDH2 inhibitors because of its high specificity and efficacy. Chemically, AVN944 is structured to bind with high affinity to the IMPDH2 enzyme, effectively shutting down its activity 16-19. This targeted approach helps to reduce the availability of guanine nucleotides, thereby hindering synthesis of DNA and RNA, both of which are essential for cancer cell proliferation. The biological advantages of AVN944 include its ability to induce G1 cell cycle arrest and promote apoptosis, mechanisms that are crucial for controlling tumor growth and progression 16, 17.

Here, we address a critical gap in treatments for Ewing's sarcoma by investigating the efficacy of AVN944. Our results indicate a clear trend: sarcoma patients with elevated levels of IMPDH2 expression have lower survival rates, reinforcing the enzyme's potential as a therapeutic target. Specifically, *in vitro* and *in vivo* tests show that AVN944 reduces cell viability and disrupts cancer cell proliferation significantly by instigating G1 cell cycle arrest and promoting apoptosis. Furthermore, *in vivo* assessments demonstrated that AVN944 effectively inhibited tumor growth in xenograft models implanted with TC71 and SK-ES-1 Ewing's sarcoma cell lines, without notable toxicity. This suggests strong therapeutic potential in a clinical setting. These findings are not only promising, but also suggest that AVN944 could serve as a cornerstone for developing more effective treatment regimens. The data procured from our studies provide compelling evidence supporting the inhibitor's role in advancing Ewing's sarcoma treatment. By focusing on the metabolic vulnerabilities of tumor cells, AVN944 offers a novel therapeutic approach that could potentially enhance patient outcomes in a demographic critically in need of innovative solutions.

## Materials and Methods

### Patient cohort and gene expression analysis

The study utilized data from The Cancer Genome Atlas (TCGA) sarcoma cohort to examine expression of IMPDH2 and its association with overall survival (OS) of sarcoma patients. Gene expression data, measured in transcripts per million (TPM), and clinical data, including survival outcomes, were downloaded from the TCGA database. Patients were stratified into high and low IMPDH2 expression groups based on the median TPM value of IMPDH2. Kaplan-Meier survival analysis was performed to compare the OS of the two groups, with hazard ratios calculated and statistical significance assessed using the log-rank test. Box plots were generated to compare IMPDH2 expression between tumor samples (n = 262) and normal tissue samples (n = 2), with expression values presented as log₂(TPM + 1).

### Cell lines and cell culture

The following human cell lines were used in this study: Ewing's sarcoma cell lines RD-ES, SK-ES-1, and TC71; HeLa (human cervical carcinoma cell line); and MCF-7 (human breast cancer cell line). The Ewing's sarcoma cell lines were selected to analyze IMPDH2 expression in sarcoma, while HeLa and MCF-7 served as positive controls because of their known high expression of IMPDH2. The cell lines RD-ES, SK-ES-1, HeLa, and MCF-7 were obtained from the American Type Culture Collection (ATCC). By contrast, the TC71 cell line was acquired from the Leibniz Institute DSMZ (Germany). All cell lines were cultured in their recommended medium: Dulbecco's Modified Eagle's Medium (DMEM) for HeLa and MCF-7; Roswell Park Memorial Institute 1640 Medium (RPMI-1640) for RD-ES and SK-ES-1; and Iscove's Modified Dulbecco's Medium for TC-71. Culture media were supplemented with 10% fetal bovine serum (ThermoFisher Scientific), GlutaMAX (ThermoFisher Scientific), and 1% penicillin-streptomycin (ThermoFisher Scientific). Cells were maintained at 37°C in a humidified incubator (ThermoFisher Scientific) under 5% CO₂.

### Western blot analysis

Equal amounts of protein lysate were loaded into each lane of an SDS-PAGE gel and separated by electrophoresis as described previously 20. Briefly, following electrophoresis, proteins were transferred onto a polyvinylidene difluoride membrane, which was then incubated with the following primary antibodies: anti-IMPDH2 (Proteintech), anti-p53 (Santa Cruz Biotechnology), anti-Cyclin D1 (Santa Cruz Biotechnology), anti-Cyclin E (Santa Cruz Biotechnology), anti-Bax (Proteintech), anti-Bcl-2 (Santa Cruz Biotechnology), anti-PARP1 (Proteintech), and anti-α/β-Tubulin (Cell Signaling Technology). Protein bands were visualized using an enhanced chemiluminescence (ECL) detection system (PerkinElmer Life Sciences), and the intensity of the protein bands was analyzed using ImageJ software (https://imagej.nih.gov/ij/). α/β-Tubulin was used to validate equal protein loading across all lanes.

### Drug treatment

AVN944 (Sigma-Aldrich) was dissolved in DMSO to prepare a stock solution, and then diluted to the desired concentrations in cell culture medium immediately before use. Control cells were treated with an equivalent volume of vehicle (DMSO). For all experiments, the final DMSO concentration did not exceed 0.1%.

### Cell growth assay

To monitor cell growth, TC71 cells were seeded in 12-well plates at a density of 1 × 10^4^ cells per well (SK-ES-1 cells were seeded at 5 × 10^4^ cells per well) and treated with 1 μM AVN944 or vehicle control. Phase-contrast microscopy images were taken daily for 5 days to assess cell morphology and density. Cells were counted using a hemocytometer to generate a growth curve for each time point. Data are represented as the mean ± standard deviation (SD). Each experiment was performed in triplicate (n = 3).

### Measurement of intracellular ATP levels

Intracellular ATP levels were quantified using an ATP Assay Kit (Abcam). TC71 and SK-ES-1 cells were treated for 24 h with 1 μM AVN944 or vehicle control (0.1% DMSO). After treatment, cells (1 × 10^6^) were collected by trypsinization, washed once with PBS, and resuspended in 100 μL ATP assay buffer. The cell suspensions were lysed and centrifuged to remove debris, and the resulting supernatants were incubated with the ATP probe provided in the kit. Absorbance was measured at 570 nm using a SpectraMax microplate reader (Molecular Devices). ATP levels were normalized to the control group (set as 100%), and data were expressed as the mean ± SD from eight independent experiments.

### BrdU incorporation assay

DNA synthesis was evaluated using a BrdU incorporation assay. TC71 or SK-ES-1 cells were seeded in 12-well plates at a density of 5 × 10^4^ cells per well, treated with 5 μM AVN944 or vehicle control for 24 h, and then incubated with 25 μM BrdU (BD Pharmingen) for 3 h. After fixation in 4% paraformaldehyde (Sigma-Aldrich), cells were stained with an anti-BrdU antibody (Abcam; green) and counterstained with DAPI (Life Technologies; blue) to visualize nuclei. Images were acquired using a Confocal microscope (Leica TSC SPE, Leica Microsystems), and BrdU-positive cells were quantified in at least three random fields per well. Data are representative of three independent experiments (n = 3).

### MTT assay

Cell viability was assessed using an MTT assay kit (Cell Proliferation Kit I, Roche). TC71 cells were seeded in 96-well plates at a density of 3.5 × 10^3^ cells (2 × 10^3^ cells for SK-ES-1 cells) per well and treated with 5 μM AVN944 or vehicle control for 3 days. After treatment, cells were incubated for 4 h at 37°C with 20 μL of 5 mg/mL MTT solution. Formazan crystals were dissolved in 100 μL DMSO, and absorbance was measured at 570 nm and 660 nm using a microplate reader (Molecular Devices). Data were normalized to the control group and presented as the mean ± SD of eight independent experiments (n = 8).

### Colony formation assay

To evaluate long-term cell proliferation, TC71 cells were seeded in six-well plates at a density of 1 × 10^3^ cells (5 × 10^3^ cells for SK-ES-1 cells) per well and then treated with AVN944 at concentrations ranging from 0 to 1 μM. TC71 or SK-ES-1 cells were cultured for 5 or 7 days, respectively, with the medium being replenished every 3 days. Colonies were fixed and stained for 30 min with a solution containing 0.05% (w/v) Crystal Violet (Sigma-Aldrich), 1% formaldehyde (Sigma-Aldrich), 1% methanol (Sigma-Aldrich), and 1 × phosphate-buffered saline (PBS). The number of colonies was quantified using ImageJ software, and colony counts were normalized to the control. Data are presented as the mean ± SD of three independent experiments (n = 3).

### Phase-contrast microscopy

Morphological changes and cell density were assessed by phase-contrast microscopy. Images were captured under an inverted microscope (IX71; Olympus). Representative images were selected from three independent experiments to evaluate the effects of AVN944 on cell morphology and proliferation.

### Determination of the half-maximal inhibitory concentration (IC_50_)

The IC_50_ of AVN944 was calculated using dose-response curves, as described previously 21. Cell viability data were fitted to a four-parameter logistic regression model using SoftMax Pro software (Molecular Devices). The R² value was used to assess the goodness of fit of the model. IC_50_ values were calculated from three independent experiments, with data presented as the mean ± SD.

### Flow cytometry analysis of the cell cycle

To evaluate cell cycle distribution, cells were harvested, washed twice with PBS, and fixed overnight in 70% ethanol at 4 °C as described previously 22. Fixed cells were stained for 30 min at 37 °C with 50 μg/mL propidium iodide (PI) solution (BioLegend) containing 100 μg/mL RNase A/PBS. Flow cytometry was performed using a flow cytometer (CytoFLEX, Beckman Coulter Life Sciences), and cell cycle phases (sub-G1, G1, S, and G2/M) were quantified.

### Apoptosis assay using Annexin V/PI staining

Apoptosis was assessed using an Annexin V-FITC/PI Apoptosis Detection Kit (BioLegend). Briefly, cells were harvested, washed twice with PBS, and resuspended in a binding buffer (BioLegend). Cells were then stained with Annexin V-FITC and PI for 15 min at room temperature in the dark. Stained cells were analyzed immediately using a flow cytometer (CytoFLEX, Beckman Coulter Life Sciences). Early apoptotic cells were defined as Annexin V-positive and PI-negative, while late apoptotic or necrotic cells were Annexin V-positive and PI-positive. Cell viability was determined by quantifying the PI- and Annexin V-negative populations.

### Xenograft tumor induction and treatment regimen

Eight-week-old male nude mice (Orient Bio Inc., Korea) were housed under specific pathogen-free conditions with a 12-h light/dark cycle and given access to food and water *ad libitum*. To establish TC71 xenografts, 2 × 10^6^ cells were injected subcutaneously into the right flank of each mouse. To establish SK-ES-1 xenografts, 4 × 10^6^ cells were injected. After the tumor volume reached approximately 15-25 mm^3^, mice were randomized into treatment groups, and AVN944 was administered intraperitoneally at a dose of 50 mg/kg every 3 days. Control groups received an equivalent volume of vehicle solution. Body weight was monitored every other day to assess systemic toxicity. Tumor volume was measured using digital calipers and calculated using the formula: volume = (length × width^2^)/2. At the end of the treatment period, mice were euthanized, and tumors were excised, weighed, and photographed for further analysis, as described previously 23. All animal experiments were conducted in accordance with institutional guidelines and approved by the Institutional Animal Care and Use Committee (IACUCSGU2024_09) of the hosting research facility.

### Statistical analysis

Microsoft Excel was utilized for all statistical evaluations. Results are expressed as the mean ± SD. Statistical significance was assessed using Student's *t*-test, with a threshold of *p* < 0.05 used to indicate significance. All experiments were conducted in triplicate unless stated otherwise to ensure reliability and consistency.

## Results

### Elevated expression of IMPDH2 correlates with a trend toward decreased survival in patients with sarcoma

**Figure 1:**
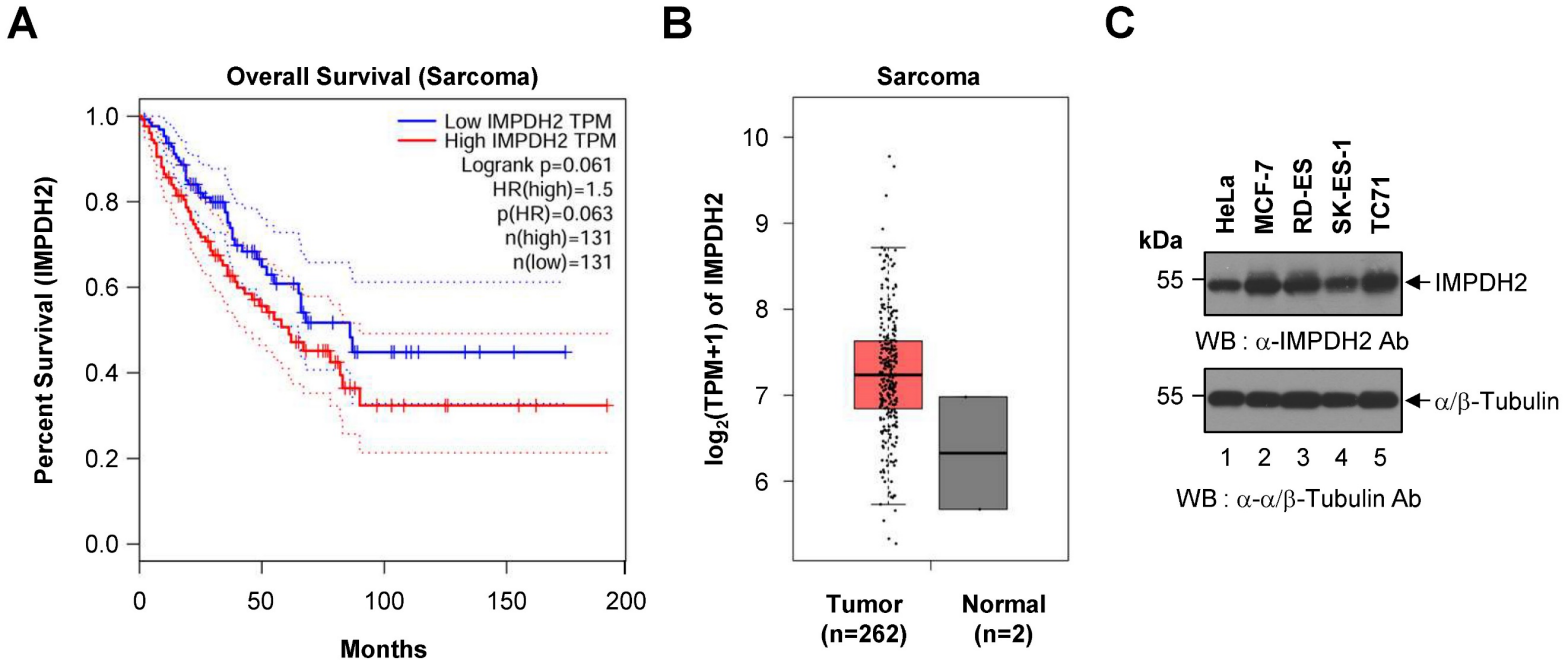
** Kaplan-Meier survival analysis and differential expression of IMPDH2 in sarcoma. (A)** Overall survival curve based on IMPDH2 expression in patients with sarcoma from the TCGA cohort (n = 262). Patients were stratified into low IMPDH2 expression (blue line) and high IMPDH2 expression (red line) groups. Kaplan-Meier curves show that higher expression of IMPDH2 is associated with a trend towards reduced survival, although this did not reach statistical significance (log-rank *p* = 0.061). The hazard ratio for high expression was 1.5, indicating a 50% increase in the risk of death for patients with high expression of IMPDH2 (*p* = 0.063).** (B)** Differential expression of IMPDH2 between sarcoma (Tumor, n = 262) and normal tissues (Normal, n = 2) from the TCGA dataset. IMPDH2 expression, measured in transcripts per million (TPM), is significantly higher in tumor samples than in normal tissues. **(C)** Western blot analysis to detect expression of IMPDH2 in Ewing's sarcoma cell lines (RD-ES, SK-ES-1, and TC71). HeLa (human cervical carcinoma cell line) and MCF-7 (human breast cancer cell line) cells were used as positive controls. α/β-tubulin served as a loading control.

To assess the prognostic relevance of IMPDH2 expression in sarcoma, we performed a Kaplan-Meier survival analysis using data from TCGA cohort. Patients were dichotomized into high and low IMPDH2 expression groups based on median TPM values. As shown in Figure 1A, patients with high expression of IMPDH2 exhibited a trend towards poorer OS than those with low expression (HR = 1.5, *p* = 0.063). Although this trend was not statistically significant (log-rank *p* = 0.061), it suggests that higher expression of IMPDH2 may be associated with decreased survival of patients with sarcoma. The dotted lines in the Kaplan-Meier plot represent the 95% confidence intervals for each survival curve, indicating the variability and uncertainty surrounding the estimated survival probabilities.

We further evaluated the clinical relevance of GTP biosynthesis-related enzymes by performing Kaplan-Meier survival analyses using TCGA sarcoma datasets. Patients were stratified into high and low expression groups based on median TPM values for each gene. Expression of the GMPR and GMPR2 genes suppresses GTP biosynthesis, while that of IMPDH1, GUK1, NME1, and GMPS promotes GTP biosynthesis, underscoring their potential functional relevance in tumor biology 13, 24. As shown in Supplementary Figure S1, among the enzymes analyzed, high expression of GMPR was associated significantly with improved overall survival of sarcoma patients (HR = 0.57, *p* = 0.006, log-rank *p* = 0.0053), suggesting a potential protective role of GMPR. By contrast, expression levels of GMPR2 (HR = 0.81, *p* = 0.33, log-rank *p* = 0.83), IMPDH1 (HR = 0.78, *p* = 0.22, log-rank *p* = 0.22), GUK1 (HR = 0.86, *p* = 0.47, log-rank *p* = 0.46), NME1 (HR = 1.3, *p* = 0.25, log-rank *p* = 0.24), and GMPS (HR = 1.1, *p* = 0.53, log-rank *p* = 0.52) did not show significant associations with overall survival. These findings suggest that while GMPR may serve as a potential prognostic biomarker for sarcoma, the prognostic value of other GTP biosynthesis-related enzymes within this dataset appears to be limited. Collectively, these analyses highlight the importance of GTP metabolism in sarcoma biology, and underscore the need for further mechanistic studies to elucidate the specific contributions of individual enzymes to progression of sarcoma.

To further investigate the role of IMPDH2 in sarcoma, we analyzed its expression levels in tumor tissues and compared them with those in normal tissues. Figure 1B demonstrates that expression of IMPDH2 was elevated markedly in sarcoma tumors (n = 262) relative to normal tissue samples (n = 2), supporting a potential oncogenic role for IMPDH2 in this cancer type.

To confirm the presence of IMPDH2 protein in Ewing's sarcoma, we conducted western blot analysis of three representative Ewing's sarcoma cell lines (RD-ES, SK-ES-1, and TC71). IMPDH2 was detected across all three cell lines, as well as in HeLa and MCF-7 cells, which served as positive controls (Figure 1C). The α/β-Tubulin bands confirmed equal loading of cell lysates in each lane, validating the observed expression levels of IMPDH2.

### AVN944 treatment inhibits growth and colony formation by TC71 Ewing's sarcoma cells

**Figure 2:**
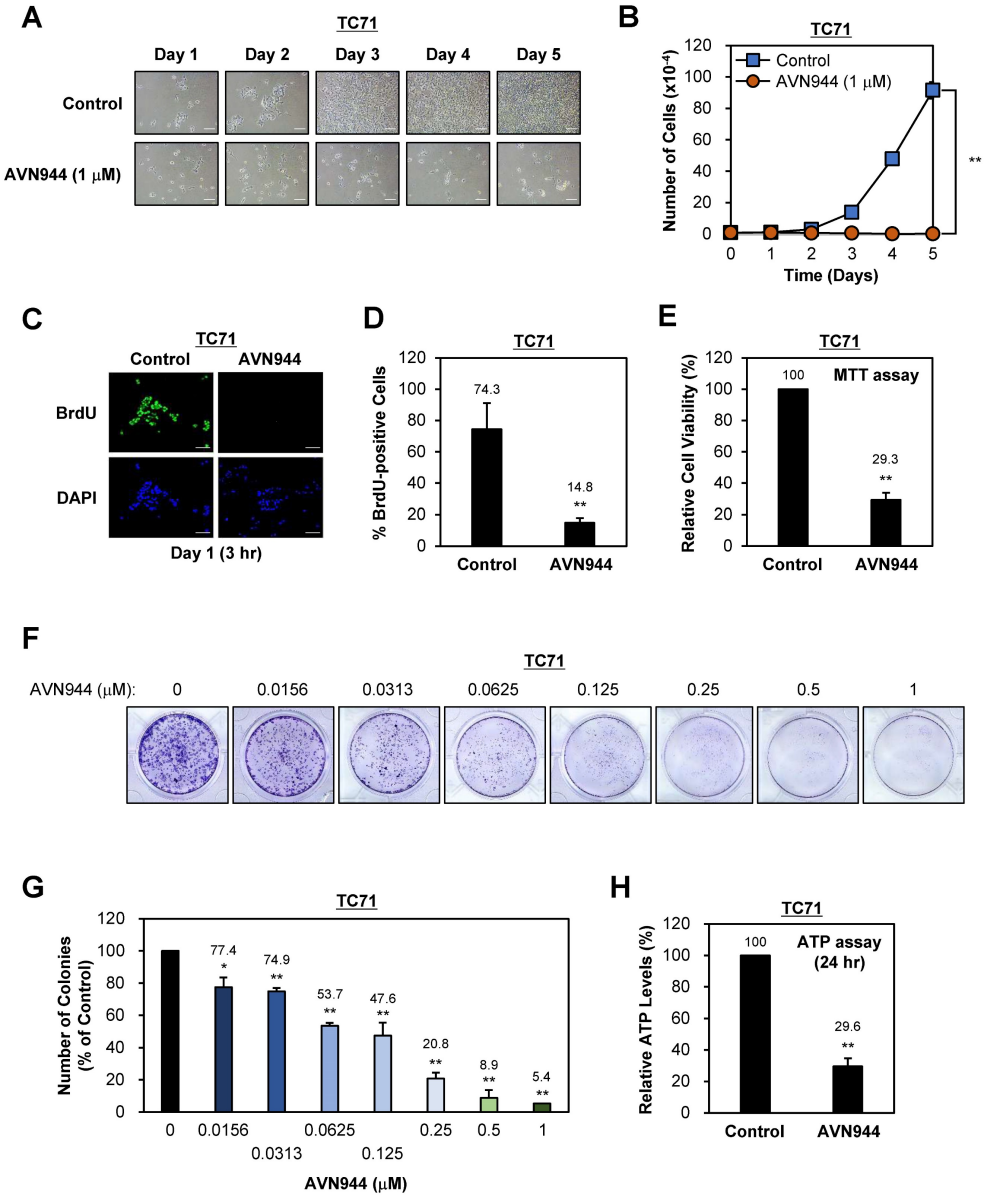
** Effects of AVN944 on growth and colony formation by TC71 Ewing's sarcoma cells. (A)** Representative images of TC71 cells treated with 1 μM AVN944 or control (vehicle) over 5 days. Morphological changes were observed, with AVN944-treated cells showing lower cell density than control cells. Scale bar, 100 μm. **(B)** Growth curve for TC71 cells treated with 1 μM AVN944 (orange) or control (blue) for 5 days. AVN944 inhibited cell proliferation significantly when compared with the control. Data represent the mean ± SD of three independent experiments (n = 3, **p < 0.01). **(C)** Representative images from a BrdU incorporation assay in which TC71 cells were treated with AVN944 for 3 h on Day 1. BrdU-positive cells (green) are actively proliferating. Nuclei were stained with DAPI (blue). Scale bar, 100 μm. **(D)** Quantification of BrdU-positive TC71 cells treated (or not) with AVN944. AVN944 reduced BrdU incorporation significantly, indicating a reduction in DNA synthesis (n = 3, **p <0.01). **(E)** MTT assay of TC71 cells treated with AVN944. AVN944 treatment resulted in a significant reduction in cell viability compared with the control (n = 8, **p <0.01). **(F)** Colony formation assay in which TC71 cells were treated with increasing concentrations of AVN944 (0-1 μM). Colonies were stained with crystal violet, which revealed dose-dependent inhibition of colony formation. **(G)** Quantification of colony formation in F. The number of colonies was counted using ImageJ software, and presented as a percentage of the control value. AVN944 reduced colony formation significantly and in a dose-dependent manner (*p < 0.05, **p < 0.01 *vs.* control). Data represent the mean ± SD of three independent experiments (n = 3). **(H) Measurement of intracellular ATP levels in TC71 cells treated with AVN944 for 24 h.** AVN944 reduced cellular ATP levels significantly compared with the control, indicating a decline in cellular metabolic activity (**p < 0.01 *vs.* control). Data represent the mean ± SD of eight independent experiments (n = 8).

To evaluate the effect of the IMPDH2 inhibitor AVN944 on proliferation of TC71 Ewing's sarcoma cells, we treated them with 1 μM AVN944 and monitored morphological changes over 5 days. Compared with control cells, AVN944-treated cells exhibited noticeable morphological alterations, including reduced cell density and changes in cell shape over time, suggesting an inhibitory effect on cell growth (Figure 2A). Daily cell counts revealed a significant reduction in proliferation of cells exposed to 1 μM AVN944, with the growth curve showing substantial suppression compared with that of the control (Figure 2B). These results indicate that AVN944 effectively inhibits TC71 cell proliferation.

To assess the impact of AVN944 on DNA synthesis, we conducted a BrdU incorporation assay after treating cells with 5 μM AVN944. The number of BrdU-positive cells, representing cells in the S phase of the cell cycle, was significantly lower in the AVN944-treated group than in the control group (Figure 2C and D). Specifically, AVN944 reduced BrdU-positive cells to 14.8%, compared with 74.3% for the control, indicating a marked reduction in DNA synthesis and suggesting that AVN944 disrupts cell cycle progression.

Next, we investigated the cytotoxic effects of AVN944 on TC71 cells by measuring cell viability in an MTT assay. Treatment with 5 μM AVN944 led to a significant reduction in cell viability, with only 29.3% of cells remaining viable compared with untreated controls (Figure 2E). This finding confirms the cytotoxic effects of AVN944 on TC71 cells, further supporting its potential as an inhibitor of Ewing's sarcoma cell survival.

The impact of AVN944 on the clonogenic potential of TC71 cells was assessed in a colony formation assay across a range of AVN944 concentrations (0 to 1 μM). Visual inspection of crystal violet-stained plates revealed a marked reduction in colony number and size as the concentration of AVN944 increased (Figure 2F). Quantitative analysis using ImageJ software confirmed dose-dependent suppression of colony formation, with significant inhibition observed at AVN944 concentrations as low as 0.0156 μM (Figure 2G). At 0.5 μM and 1 μM, colony formation was almost abolished, indicating potent inhibition of long-term cell survival and self-renewal capacity by AVN944.

To further assess the impact of AVN944 on cellular metabolic activity, we measured intracellular ATP levels in TC71 cells following treatment with AVN944 for 24 h. AVN944 caused a significant reduction in ATP levels, with treated cells exhibiting a level only 29.6% that in control cells (Figure 2H). This decrease in the amount of ATP indicates impaired cellular metabolic activity upon treatment with AVN944, further supporting its cytotoxic and anti-proliferative effects.

### AVN944 suppresses the clonogenic potential of SK-ES-1 cells in a dose-dependent manner

**Figure 3:**
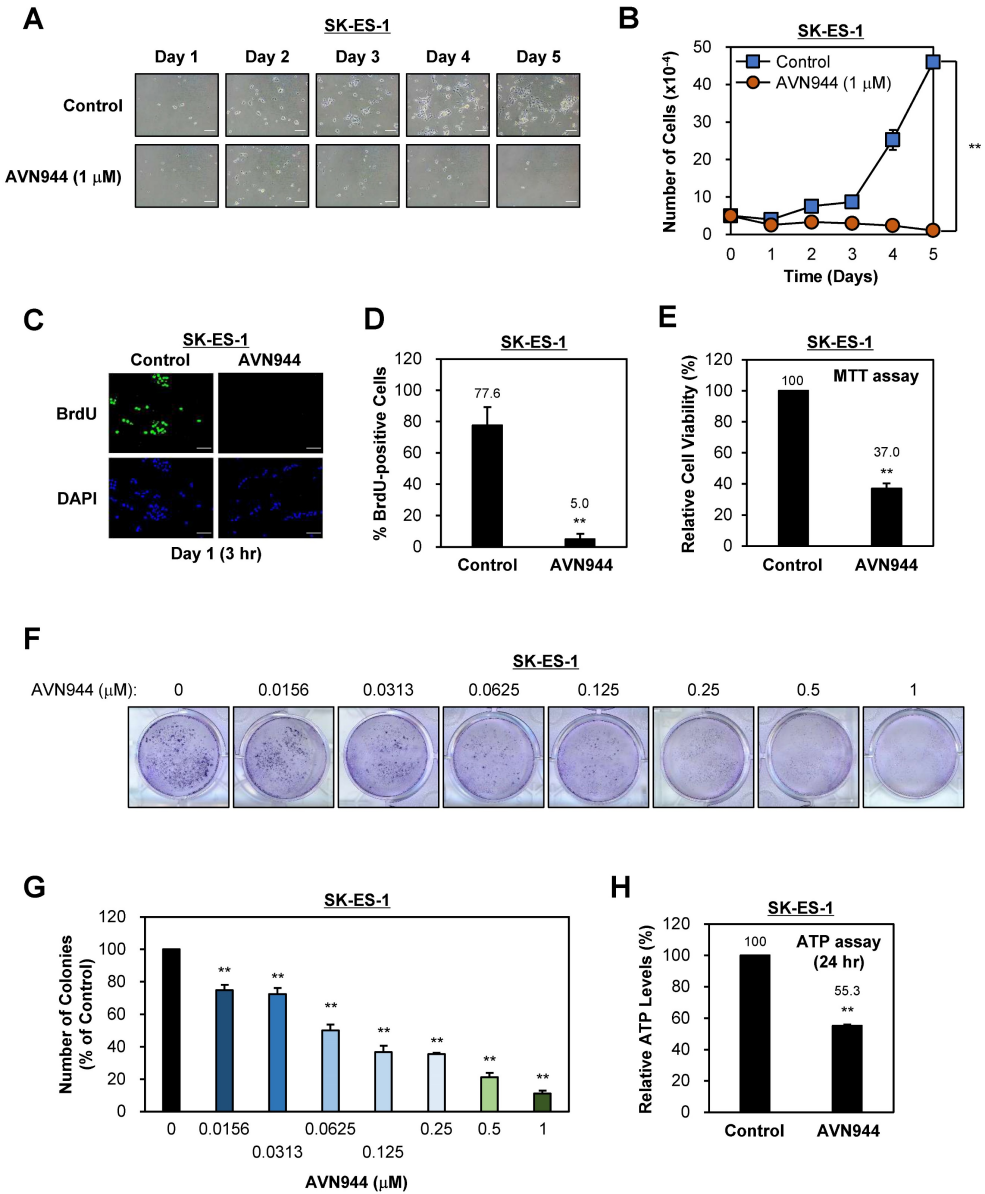
** Effects of AVN944 on growth and colony formation by SK-ES-1 Ewing's sarcoma cells. (A)** Representative images of SK-ES-1 cells treated with 1 μM AVN944 or control (vehicle) over 5 days. The density of cells treated with AVN944 was lower than that of cells treated with the control. Scale bar, 100 μm. **(B)** Growth curve of SK-ES-1 cells treated with 1 μM AVN944 (orange) or control (blue) for 5 days. AVN944 inhibited proliferation of SK-ES-1 cells significantly when compared with the control (n = 3, **p < 0.01). (C) Immunofluorescence images of BrdU-stained SK-ES-1 cells treated with AVN944 for 3 h on Day 1. DAPI was used to stain the nuclei. Scale bar, 100 μm.** (D)** Quantification of BrdU-positive SK-ES-1 cells treated with AVN944. Data represent the mean ± SD; **p < 0.01 (n=3). **(E)** MTT assay showing the relative cell viability of SK-ES-1 cells treated with AVN944 (compared with the control). Data represent the mean ± SD; **p < 0.01 (n = 8). **(F)** Colony formation assay for SK-ES-1 cells treated with various concentrations of AVN944 (0-1 μM). Colony formation was reduced in a dose-dependent manner following AVN944 treatment, as indicated by crystal violet staining.** (G)** Quantification of colony formation in **(F)**, in which colony numbers were counted using ImageJ and are presented as a percentage of the control value. AVN944 inhibited colony formation significantly and in a dose-dependent manner (**p < 0.01 *vs.* control). Data represent the mean ± SD of three independent experiments (n = 3). **(H) Measurement of intracellular ATP levels in SK-ES-1 cells treated with AVN944 for 24 h.** AVN944 reduced cellular ATP levels significantly compared with the control, indicating a decline in cellular metabolic activity (**p < 0.01 vs. control). Data represent the mean ± SD of eight independent experiments (n = 8).

To determine the effects of AVN944 on proliferation of SK-ES-1 Ewing's sarcoma cells, we treated them with 1 μM AVN944 or vehicle control for 5 days and monitored morphology and growth. As shown in Figure 3A, AVN944 caused a marked reduction in cell density when compared with the control. The growth curve in Figure 3B supports this observation, showing significantly lower cell counts in the AVN944-treated group by Day 5 (**p < 0.01, n = 3).

To assess the impact of AVN944 on DNA synthesis, we analyzed BrdU incorporation by tumor cells. Immunofluorescence staining demonstrated a marked reduction in BrdU-positive cells upon treatment with 5 μM AVN944 (Figure 3C). Quantitative analysis revealed a marked reduction in BrdU incorporation, from 77.6% in control SK-ES-1 cells to 5.0% in AVN944-treated cells (Figure 3D, **p < 0.01).

Cell viability was assessed in an MTT assay, the results of which showed that 5 μM AVN944 reduced cell viability to 37.0% compared with that of untreated controls (Figure 3E, **p < 0.01).

To evaluate the impact of AVN944 on the clonogenic ability of SK-ES-1 cells, we performed a colony formation assay. Figure 3F shows that AVN944 inhibited colony formation in a dose-dependent manner, with increasing concentrations of AVN944 (0.0156-1 μM) resulting in fewer colonies than observed for the control. Quantification of colony formation, as shown in Figure 3G, revealed significant inhibition at concentrations as low as 0.0156 μM (*p* < 0.01). At 1 μM, near-complete inhibition of colony formation was observed.

To further investigate the impact of AVN944 on cellular metabolic activity, we measured intracellular ATP levels in SK-ES-1 cells following treatment with AVN944 for 24 h. AVN944 caused a significant reduction in ATP levels, with treated cells retaining only 55.3% of the levels observed in control cells (Figure 3H, *p* < 0.01). This reduction indicates impaired cellular metabolic activity upon treatment with AVN944, further supporting its cytotoxic and anti-proliferative effects.

### IMPDH2 inhibitors mycophenolic acid (MPA) and sappanone A (SA) suppress proliferation of, and colony formation by, Ewing's sarcoma cells

To confirm that the inhibitory effects of AVN944 are attributable to blockade of IMPDH2 activity rather than compound-specific actions, we next tested the effects of two structurally and mechanistically distinct IMPDH2 inhibitors, MPA and SA, against Ewing's sarcoma cell lines TC71 and SK-ES-1.

Treatment of TC71 cells with 1 μM MPA or SA reduced cell density markedly over 5 days, as visualized by phase-contrast microscopy, indicating impaired cell proliferation. Growth curve analysis revealed significant suppression of proliferation relative to the control (*p* < 0.01), paralleling the effects of AVN944. Colony-formation assays further demonstrated a dose-dependent reduction in clonogenic potential across a concentration range of 0-1 μM. Quantitative analysis showed that MPA reduced colony numbers by more than 80% at ≥ 0.5 μM, whereas SA abolished colony formation almost completely at ≥ 0.25 μM (*p* < 0.01) (Supplementary Figures S2 and S3).

Similarly, treatment of SK-ES-1 cells with MPA and SA elicited antiproliferative effects, although the magnitude of inhibition differed between the two compounds. MPA led to strong suppression of cell proliferation, with markedly reduced cell counts after 5 days compared with vehicle-treated controls (*p* < 0.01). By contrast, SA induced a relatively weaker antiproliferative response under the same conditions, with only a partial reduction in cell growth. Consistent with these findings, colony-formation assays demonstrated that both inhibitors reduced clonogenic growth of SK-ES-1 cells in a dose-dependent manner; however, MPA abolished colony formation almost completely at 1 μM, whereas SA exerted a more moderate inhibitory effect, leaving a small fraction of surviving colonies even at the highest concentration tested (*p* < 0.01; Supplementary Figures S4 and S5).

### AVN944 is more cytotoxic to Ewing's sarcoma cells than to normal human cells

To assess whether AVN944 targets malignant cells selectively, we compared its effects on Ewing's sarcoma cell lines (TC71 and SK-ES-1) with those on normal human cells (i.e., normal lung fibroblasts (MRC-5), human mesenchymal stem cells (MSCs), and human dermal fibroblasts (HDFs)). After 5 days of exposure to 1 μM AVN944, proliferation of TC71 and SK-ES-1 was suppressed markedly, retaining only 0.2% and 2.3% of the control cell number, respectively (*p* < 0.01). By contrast, viability of normal cells remained substantially higher, with MRC-5, MSC, and HDF cells showing 34.0%, 10.8%, and 9.4% of control levels (*p* < 0.01; Supplementary Figure S6). These findings indicate that normal cells are relatively resistant to AVN944 concentrations that strongly suppress Ewing's sarcoma cells, demonstrating a clear therapeutic window, as well as the tumor selectivity, of AVN944. This selectivity suggests that AVN944 is a promising candidate for targeted therapy of Ewing's sarcoma.

### Dose-dependent inhibition of TC71 cells by AVN944

**Figure 4:**
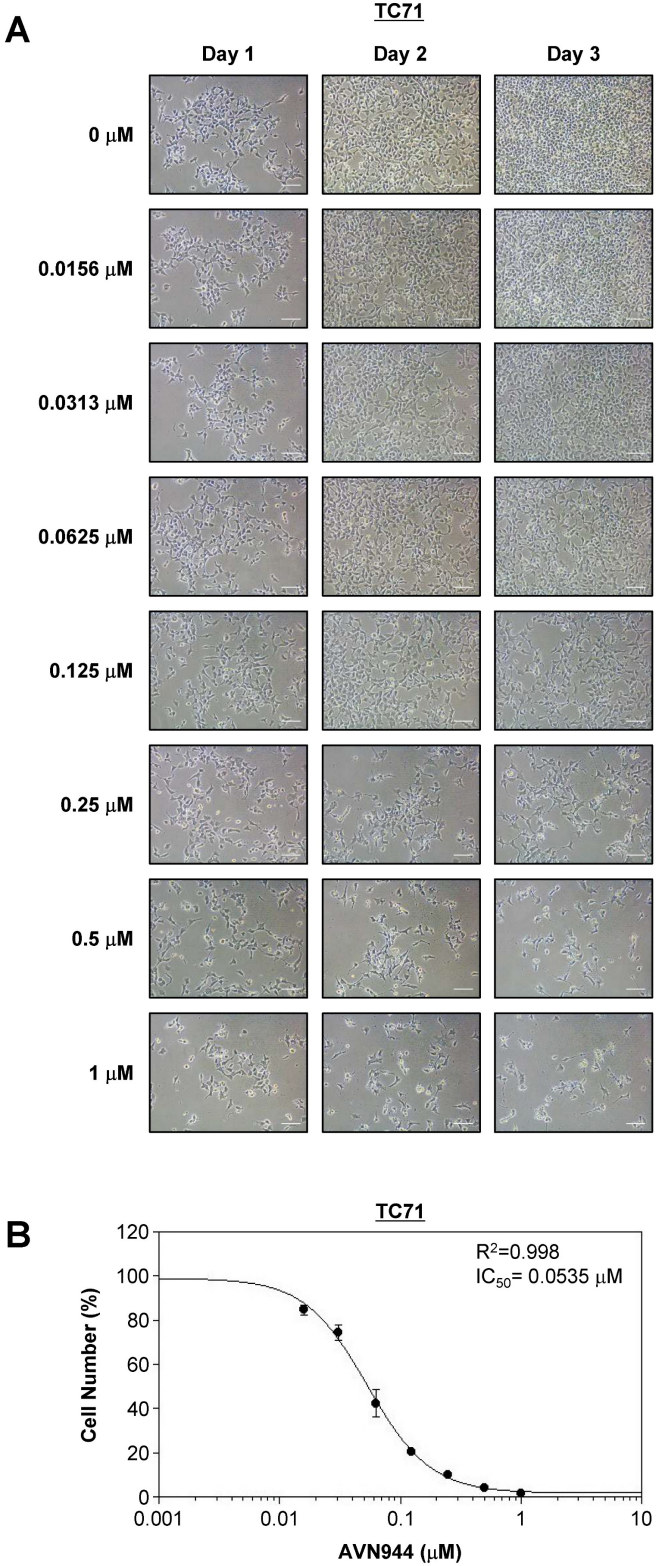
** Dose-dependent inhibition of TC71 cell proliferation by AVN944, and determination of the IC_50_. (A)** Representative phase-contrast images of TC71 cells treated with varying concentrations of AVN944 (0-1 μM) for 3 days. Cells were imaged on Days 1, 2, and 3 post-treatment. AVN944 caused a concentration-dependent reduction in cell density, as well as changes in cell morphology, particularly at higher concentrations. Scale bar, 100 μm.** (B)** Dose-response curve for TC71 cells treated with AVN944 for 3 days. Cell viability was measured, and the half-maximal inhibitory concentration (IC_50_) was calculated as 0.0535 μM, with an R² value of 0.998, indicating a solid fit of the data to the model. Data are presented as the mean ± SD of three independent experiments (n = 3).

To determine the inhibitory concentration at which 50% of TC71 cells are affected by AVN944 (i.e., the IC_50_), cells were treated with increasing concentrations (0-1 μM) of the drug for 3 days, during which cell morphology and growth were monitored. Figure 4A shows phase-contrast images of TC71 cells treated with various concentrations of AVN944 over 3 days. Morphological changes, including reduced cell density and increased cell death, were dose-dependent, with more pronounced at concentrations of 0.125 μM and above.

Cell viability was quantified on Day 3, and a dose-response curve was generated to calculate the IC_50_ value. As shown in Figure 4B, AVN944 inhibited TC71 cell proliferation in a dose-dependent manner, with an IC_50_ of 0.0535 μM. The R² value of 0.998 suggests a strong correlation between AVN944 concentration and inhibitory effects on cell growth. Thus, AVN944 is highly effective at inhibiting proliferation of TC71 cells at low micromolar concentrations.

### AVN944 inhibits SK-ES-1 cell growth in a dose-dependent manner

**Figure 5:**
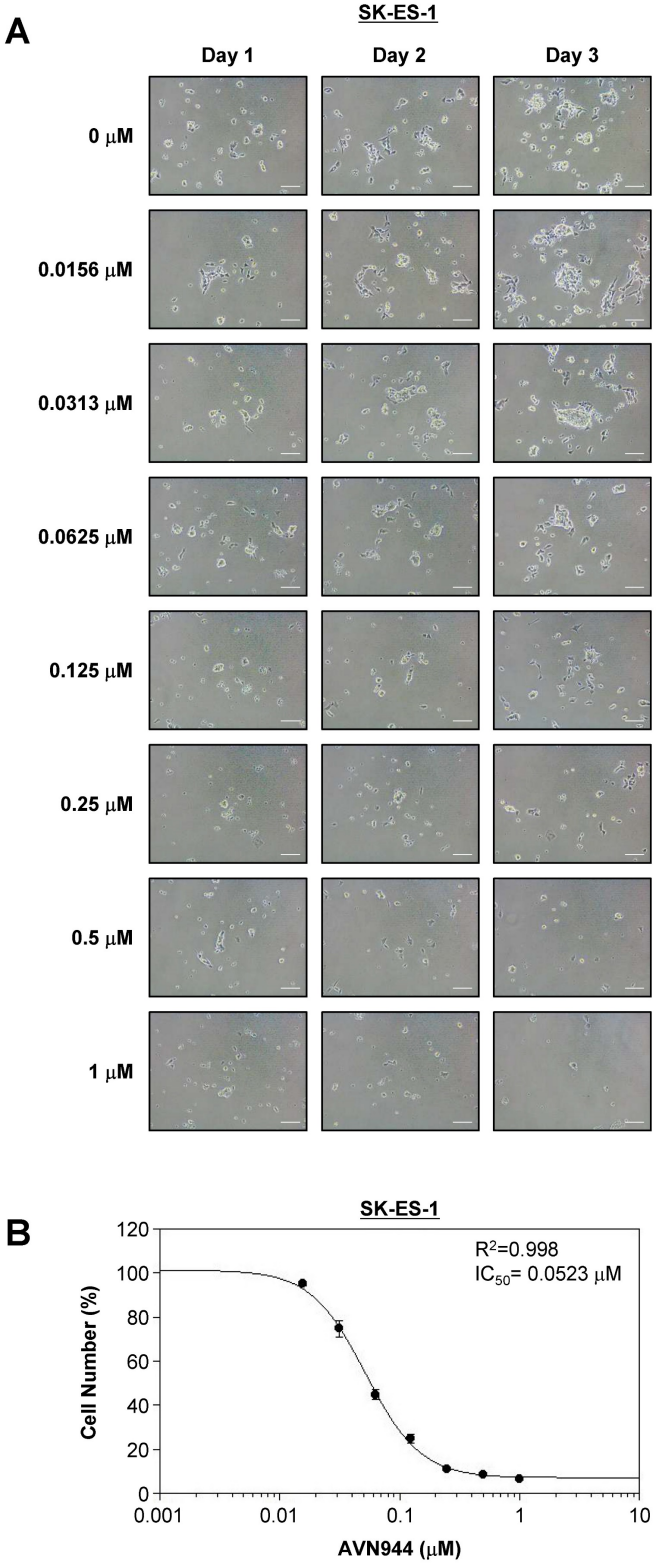
** Dose-dependent inhibition of SK-ES-1 cell proliferation by AVN944, and determination of the IC_50_. (A)** Representative phase-contrast images of SK-ES-1 cells treated with increasing concentrations of AVN944 (0-1 μM) for 3 days. Morphological changes were observed, with AVN944 treatment causing a concentration-dependent decrease in cell density, especially at higher concentrations. Images were taken on Days 1, 2, and 3 post-treatment. Scale bar, 100 μm.** (B)** Dose-response curve of SK-ES-1 cells treated with AVN944 for 3 days, showing a dose-dependent reduction in cell viability. The half-maximal inhibitory concentration (IC_50_) was calculated as 0.0523 μM, with an R² value of 0.998, indicating a strong correlation between AVN944 concentration and its inhibitory effect. Data represent the mean ± SD of three independent experiments (n = 3).

To assess the inhibitory effects of AVN944 on SK-ES-1 Ewing's sarcoma cells, they were treated with various concentrations of AVN944 (0-1 μM) for 3 days, during which cell morphology and growth were monitored. Figure 5A shows representative images of SK-ES-1 cells treated with different concentrations of AVN944 over 3 days. Cells treated with higher concentrations of AVN944 (0.125 μM and above) exhibited a marked reduction in cell density and apparent changes in morphology, with fewer cells and signs of cell death.

Cell viability was quantified on Day 3, and a dose-response curve was generated to determine the IC_50_ value. As shown in Figure 5B, AVN944 inhibited SK-ES-1 cell proliferation in a dose-dependent manner, with an IC_50_ of 0.0523 μM. The R² value of 0.998 reflects a highly consistent inhibitory effect of AVN944 on SK-ES-1 cell proliferation. Thus, AVN944 effectively inhibits growth of SK-ES-1 cells at low micromolar concentrations.

### AVN944 induces apoptosis in TC71 cells

**Figure 6:**
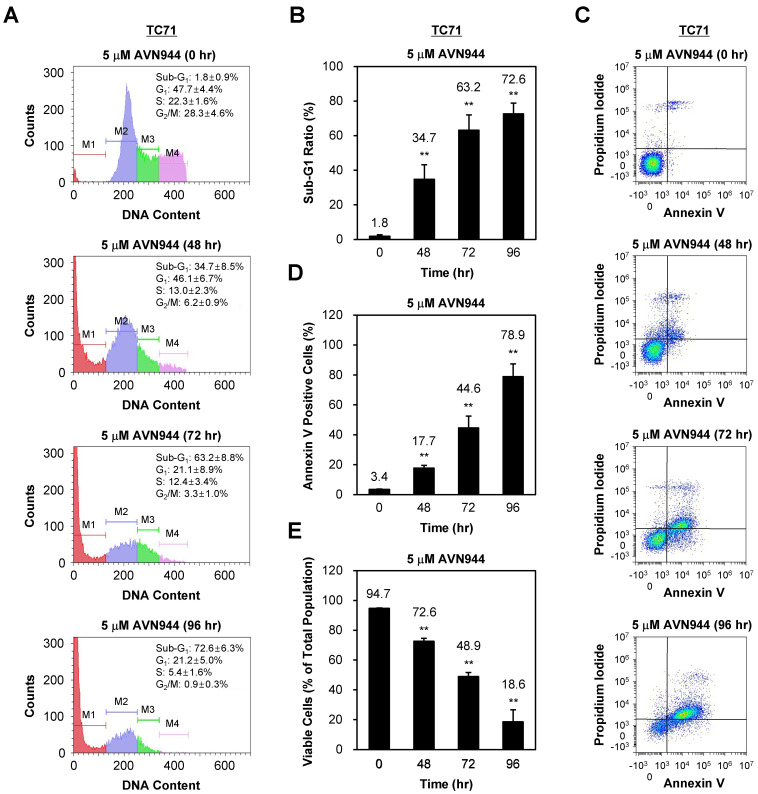
** Time-dependent induction of apoptosis of TC71 Ewing's Sarcoma cells by. AVN944. (A)** Flow cytometry analysis of DNA content in PI-stained TC71 cells treated with 5 μM AVN944 for 0, 48, 72, and 96 h. The sub-G1 (M1), G1 (M2), S phase (M3), and G2/M phase (M4) populations are indicated, along with a time-dependent increase in the sub-G1 population, indicative of apoptotic cells. **(B)** Measurement of the sub-G1 population at 48, 72, and 96 h post-treatment with AVN944. Data are presented as the mean ± SD from three independent experiments (n = 3, **p < 0.01). **(C)** Flow cytometry analysis of apoptosis (using Annexin V and PI staining) in TC71 cells treated with 5 μM AVN944 for the indicated times. Annexin V-positive cells exhibit early-stage apoptosis, while PI-positive/Annexin V-negative cells are necrotic. An apparent time-dependent increase in the percentage of Annexin V-positive cells indicates progression from early apoptosis at 48 h to advanced apoptosis by 72 and 96 h. **(D)** Quantification of Annexin V-positive apoptotic cells over time. A time-dependent increase in Annexin V-positive cells was observed, with significant elevations at 48, 72, and 96 h, indicating progression of apoptosis. By 96 h, more than 78% of TC71 cells were Annexin V-positive, confirming marked induction of apoptosis (**p < 0.01). Data are presented as the mean ± SD from three independent experiments (n=3). **(E)** Percentage of viable cells over time post-treatment with AVN944. Viable cell populations decreased significantly at 48, 72, and 96 h (**p < 0.01). Data are presented as the mean ± SD from three independent experiments (n=3).

To investigate whether AVN944 induces apoptosis in TC71 cells, we analyzed DNA content and apoptotic markers over 96 h following treatment with 5 μM AVN944. Figure 6A shows the DNA content profiles of treated cells, in which the sub-G1 population (indicative of apoptotic cells) increased progressively over time. The percentage of cells in the sub-G1 phase increased from 1.8% at 0 h to 72.6% at 96 h. Quantification of the sub-G1 population confirmed a significant increase in apoptotic cells at 48, 72, and 96 h post-treatment (Figure 6B, *p* < 0.01).

Annexin V and PI staining were performed confirm apoptosis, followed by flow cytometry analysis. As shown in Figure 6C, the percentage of Annexin V-positive cells, indicative of early apoptosis, increased over time post-AVN944 treatment. Figure 6D demonstrates a significant rise in the percentage of apoptotic cells, with 78.9% of cells being Annexin V-positive by 96 h (*p* < 0.01).

In parallel, the percentage of viable cells was assessed (Figure 6E). Treatment with AVN944 led to a significant reduction in the percentage of viable cells (from 94.7% at 0 h to 18.6% by 96 h; *p* < 0.01), suggesting that AVN944 induces apoptosis in TC71 cells in a time-dependent manner, as evidenced by the increase in the sub-G1 population and the percentage of Annexin V-positive cells.

### AVN944 triggers apoptosis in SK-ES-1 cells

**Figure 7:**
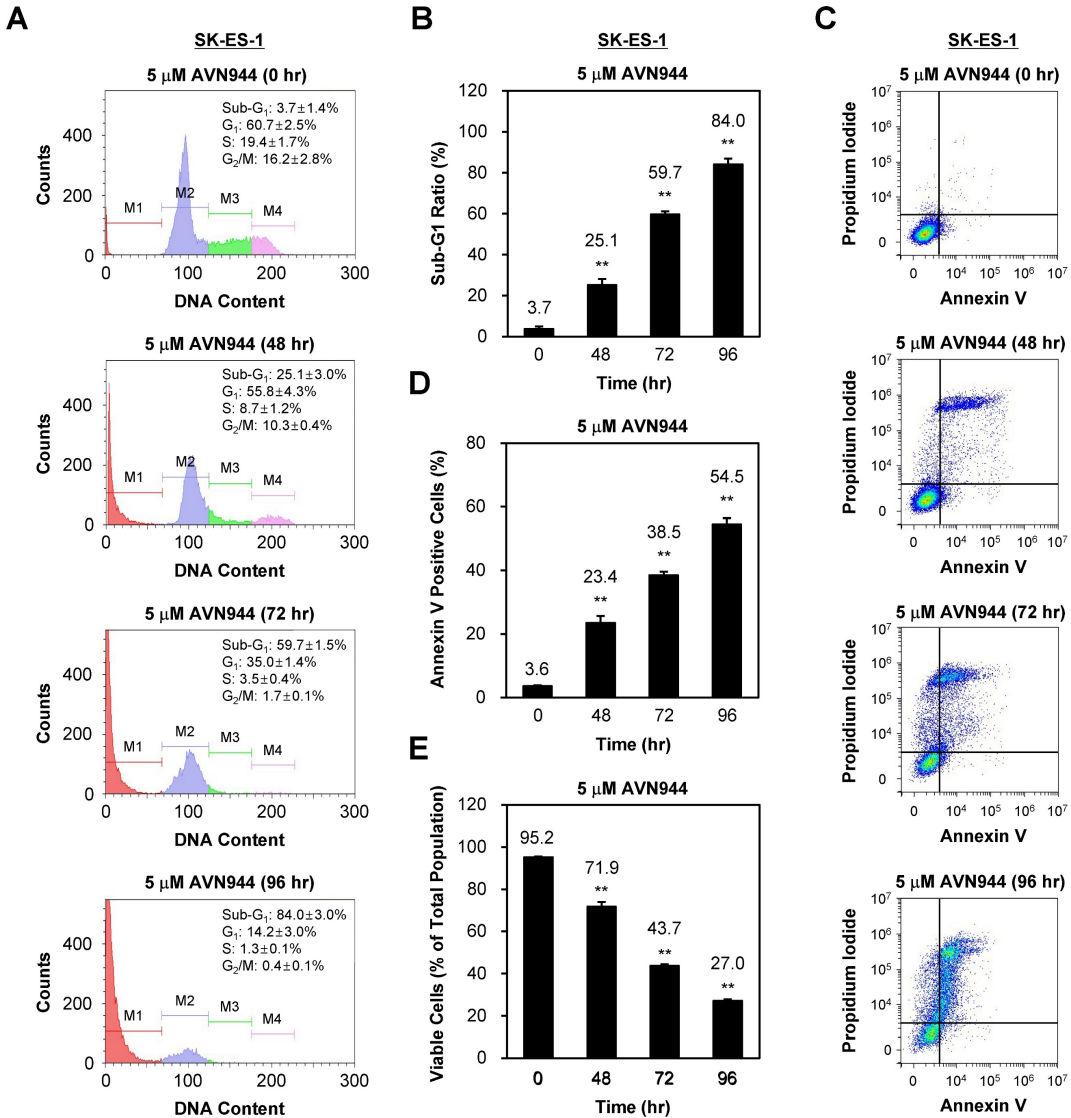
** AVN944 induces apoptosis of SK-ES-1 cells**. **(A)** Flow cytometry analysis of SK-ES-1 cells treated with 5 μM AVN944. The DNA content profiles show distinct phases of the cell cycle: sub-G1 (M1), G1 (M2), S phase (M3), and G2/M (M4). A significant increase in the sub-G1 population, indicative of apoptotic cells, was observed at 48, 72, and 96 h post-treatment, demonstrating the time-dependent effect of AVN944 cell death induction.** (B)** Quantification of the sub-G1 population at 48, 72, and 96 h post-treatment with AVN944, showing a significant increase over time. Quantifying the sub-G1 population at each time point confirmed a significant rise in the percentage of apoptotic cells, with the sub-G1 percentage increasing from 3.7% at 0 h to 84.0% at 96 h. This indicates that most cell populations underwent apoptosis following AVN944 treatment (n = 3, **p < 0.01). **(C)** Flow cytometry analysis of apoptosis (using Annexin V and PI staining) of SK-ES-1 cells treated with 5 μM AVN944 at the indicated time points. Annexin V staining detects early apoptosis, while PI staining detects late apoptosis and necrosis. As treatment progressed, there was a significant increase in the percentage of Annexin V-positive cells, confirming the apoptotic effects of AVN944.** (D)** Quantification of Annexin V-positive apoptotic SK-ES-1 cells over time. The percentage of Annexin V-positive cells increased significantly over time, reaching 54.5% by 96 h post-treatment (**p < 0.01). This further confirms that AVN944 induces apoptosis of SK-ES-1 cells in a time-dependent manner (n=3).** (E)** Percentage of viable SK-ES-1 cells at the same time points. The percentage of viable cells, determined by flow cytometry, decreased significantly over time. At 96 h, only 27.0% of the cell population remained viable, in line with the increase in apoptotic cells (**p < 0.01). This indicates a robust apoptotic response to AVN944 treatment. Data are presented as the mean ± SD from three independent experiments (n=3).

To assess whether AVN944 induces apoptosis in SK-ES-1 cells, we also performed a time-course analysis of DNA content, as well as expression of apoptotic markers, over 96 h following treatment with 5 μM AVN944. Figure 7A shows the DNA content of SK-ES-1 cells treated with AVN944, and that the sub-G1 population increased significantly over time (from 3.7% at 0 h to 84.0% by 96 h). Quantifying the sub-G1 ratio in Figure 7B demonstrates a significant time-dependent increase in the apoptotic cell population at 48, 72, and 96 h post-treatment (*p* < 0.01).

To confirm apoptosis, we also performed Annexin V and PI staining followed by flow cytometry analysis. Figure 7C shows the increase in the percentage of Annexin V-positive cells over time, indicating induction of apoptosis. Quantification of Annexin V-positive cells revealed a significant increase, reaching 54.5% by 96 h (*p* < 0.01; Figure 7D).

The percentage of viable cells decreased significantly as apoptosis progressed, from 95.2% at 0 h to 27.0% by 96 h (*p* < 0.01; Figure 7E). These results suggest that AVN944 induces apoptosis in SK-ES-1 cells in a time-dependent manner, as demonstrated by the increase in the percentages of sub-G1 and Annexin V-positive cells, and corresponding reductions in cell viability.

### AVN944 induces time-dependent changes in cell cycle regulators and apoptosis-associated proteins in TC71 cells

**Figure 8:**
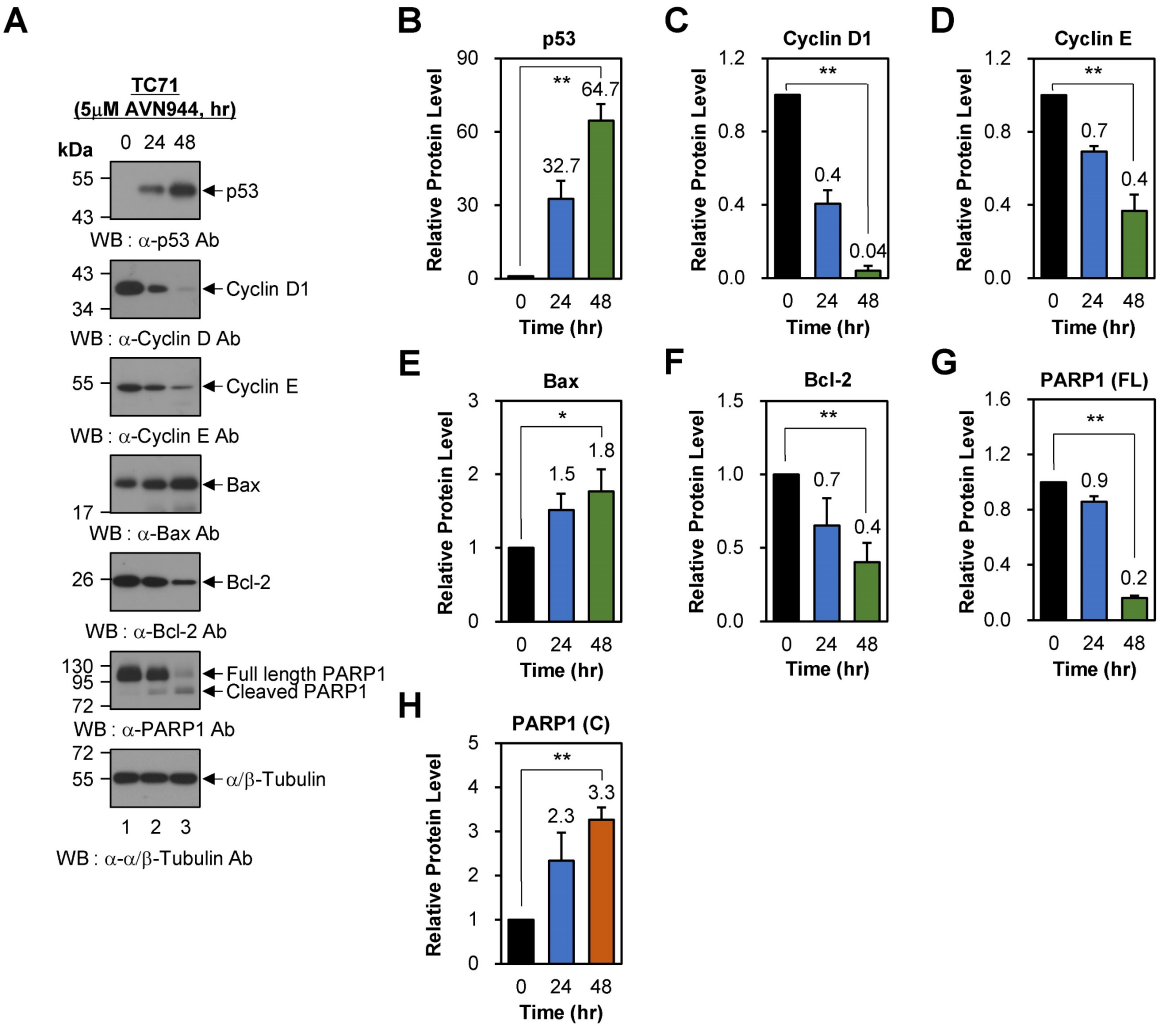
** AVN944 modulates expression of cell cycle regulators and apoptosis-associated proteins in TC71 cells. (A)** Western blot analysis of p53, Cyclin D1, Cyclin E, Bax, Bcl-2, and PARP1 (full-length and cleaved forms) expression in TC71 cells treated with 5 μM AVN944 for 0, 24, and 48 h. α/β-Tubulin was used as a loading control. AVN944 treatment led to a time-dependent increase in expression of p53, Bax, and cleaved PARP1, while there was a marked reduction in expression of Cyclin D1, Cyclin E, Bcl-2, and full-length PARP1. **(B-H)** Quantification of protein levels, normalized to α/β-Tubulin, using ImageJ software. Protein levels were measured using ImageJ software, normalized to α/β-tubulin, and further normalized to the 0-h time point for each condition. The resulting -fold changes relative to 0-h were annotated directly on the graphs to facilitate quantitative interpretation. The graphs depict relative levels of **(B)** p53, **(C)** Cyclin D1,** (D)** Cyclin E, **(E)** Bax, **(F)** Bcl-2, **(G)** full-length PARP1, and **(H)** cleaved PARP1. Data are presented as the mean ± SD of three independent experiments (n=3). Statistical significance is indicated (*p < 0.05 and **p < 0.01).

To investigate the effects of AVN944 on cell cycle regulation in Ewing's sarcoma TC71 cells, we analyzed expression of key regulatory proteins p53, Cyclin D1, and Cyclin E, following treatment with 5 μM AVN944 for 0, 24, and 48 h. Western blot analysis revealed a significant increase in p53 levels over time, with a 32.7-fold increase observed at 24 h and a 64.7-fold increase in expression of these markers at 48 h (Figures 8A and B, *p* < 0.01). By contrast, expression of Cyclin D1 and Cyclin E, both critical for the G1 to S phase transition, fell significantly. Cyclin D1 levels decreased by 60% (relative protein level = 0.4) and 96% (relative protein level = 0.04) at 24 and 48 h, respectively (Figures 8A and C; *p* < 0.01). Similarly, Cyclin E levels fell by 30% (relative protein level = 0.7) at 24 h and by 60% (relative protein level = 0.4) at 48 h (Figures 8A and 8D; *p* < 0.01). These findings indicate that AVN944 disrupts cell cycle progression of TC71 Ewing's sarcoma cells by downregulating cyclins essential for cell cycle progression.

To further understand the apoptotic effects of AVN944, we evaluated expression of key apoptotic markers Bax, Bcl-2, and PARP1. Bax, a pro-apoptotic protein involved in mitochondrial outer membrane permeabilization, showed a time-dependent increase in expression, with levels rising 1.5-fold at 24 h and 1.8-fold at 48 h (Figures 8A and E; *p* < 0.05). Conversely, the anti-apoptotic protein Bcl-2 exhibited a significant reduction in expression, decreasing by 30% (relative protein level = 0.7) at 24 h and by 60% (relative protein level = 0.4) at 48 h (Figures 8A and F; *p* < 0.01). The resulting increase in the Bax/Bcl-2 ratio favors apoptosis, suggesting that AVN944 shifts the balance towards a pro-apoptotic state in TC71 Ewing's sarcoma cells. Consistent with these findings, AVN944 induced cleavage of PARP1, a hallmark of apoptosis. Western blot analysis showed a time-dependent reduction in expression of full-length PARP1, accompanied by a corresponding increase in cleaved PARP1 levels (Figures 8A, G, and H; *p* < 0.01). At 48 h, cleaved PARP1 levels increased by 3.3-fold, while those of full-length PARP1 decreased by 80% (relative protein level = 0.2). This pattern is strongly indicative of activation of caspase-mediated apoptotic pathways.

### AVN944 induces cell cycle arrest and apoptosis in SK-ES-1 cells

**Figure 9:**
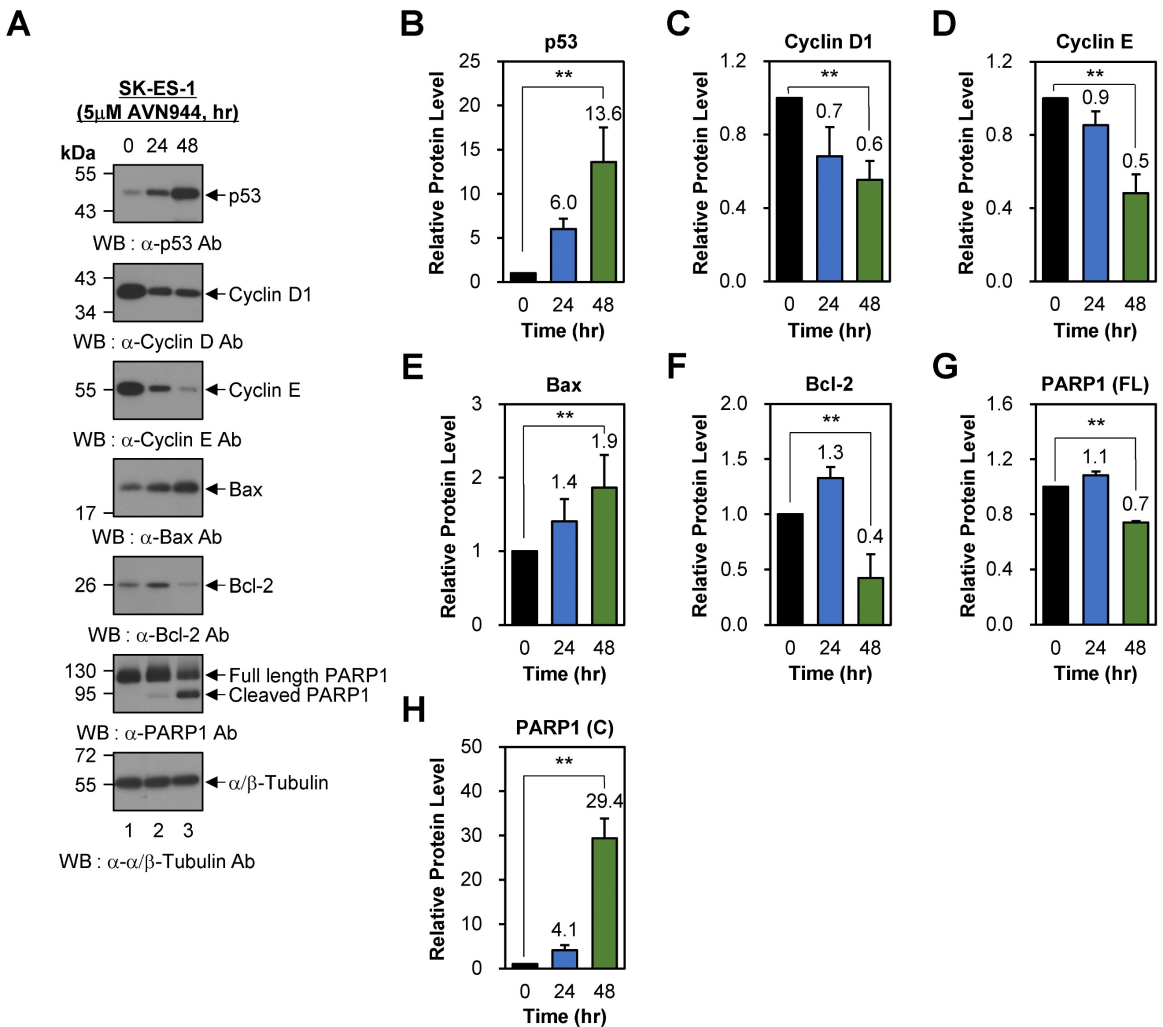
** Effects of AVN944 on cell cycle- and apoptosis-associated proteins in SK-ES-1 cells. (A)** Western blot analysis of p53, Cyclin D1, Cyclin E, Bax, Bcl-2, and PARP1 (full-length and cleaved forms) expression by SK-ES-1 cells treated with 5 μM AVN944 for 0, 24, and 48 h. AVN944 caused time-dependent upregulation of p53, Bax, and cleaved PARP1, while at the same time leading to a significant reduction in levels of Cyclin D, Cyclin E, Bcl-2, and full-length PARP1. α/β-Tubulin was used as the internal loading control. **(B-H)** Quantification of protein levels, normalized to α/β-Tubulin, using ImageJ software. Protein levels were measured using ImageJ software, normalized to α/β-tubulin, and further normalized to the 0-h time point for each condition. The resulting -fold changes relative to 0-h were annotated directly on the graphs to facilitate quantitative interpretation. Graphs depict relative expression levels of **(B)** p53, **(C)** Cyclin D1, **(D)** Cyclin E, **(E)** Bax, **(F)** Bcl-2, **(G)** full-length PARP1, and **(H)** cleaved PARP1. Data represent the mean ± SD of three independent experiments (n=3), with statistically significant changes compared with untreated controls marked (**p < 0.01).

To examine the effects of AVN944 on cell cycle regulation in SK-ES-1 cells, we also assessed the expression of p53, Cyclin D1, and Cyclin E following treatment with 5 μM AVN944 for 0, 24, and 48 h. Western blot analysis demonstrated a time-dependent increase in p53 levels, from 6.0-fold at 24 h to 13.6-fold at 48 h relative to that in untreated controls (Figures 9A and B; *p* < 0.01). By contrast, expression of Cyclin D1 and Cyclin E fell significantly over time, by 30% (relative protein level = 0.7) and 40% (relative protein level = 0.6) at 24 and 48 h, respectively (Figures 9A and C; *p* < 0.01). Similarly, Cyclin E levels declined by 10% (relative protein level = 0.9) at 24 h, and by 50% (relative protein level = 0.5) at 48 h (Figures 9A and D; *p* < 0.01). These reductions suggest that AVN944 disrupts G1-to-S phase transition in SK-ES-1 cells by inhibiting cyclin-dependent processes, thereby impairing cell proliferation.

To further investigate the pro-apoptotic effects of AVN944 in SK-ES-1 cells, we analyzed changes in Bax, Bcl-2, and PARP1 protein levels. Bax was upregulated significantly after AVN944 (by 1.4-fold at 24 h and 1.9-fold at 48 h compared with untreated controls; Figures 9A and E; *p* < 0.01). By contrast, the anti-apoptotic protein Bcl-2 exhibited a marked reduction in expression. Bcl-2 levels increased temporarily by 30% (relative protein level = 1.3) at 24 h, but fell by 60% (relative protein level = 0.4) at 48 h (Figures 9A and F; *p* < 0.01). The concurrent upregulation of Bax and downregulation of Bcl-2 shifted the Bax/Bcl-2 ratio significantly in favor of apoptosis, thereby reinforcing the role of AVN944 in promoting programmed cell death in SK-ES-1 Ewing's sarcoma cells. Consistent with these findings, AVN944 also induced cleavage of PARP1. The levels of cleaved PARP1 increased markedly over time (a 29.4-fold increase at 48 h), while full-length PARP1 levels declined by 30% (relative protein level = 0.7) over the same period (Figures 9A, G, and H; *p* < 0.01).

### Low-dose AVN944 (1 μM) recapitulates the apoptotic and cell-cycle inhibitory effects observed at higher concentrations

To verify that the cellular effects of AVN944 are not limited to high concentrations, we analyzed TC71 and SK-ES-1 cells treated with 1 μM AVN944 for up to 96 h. Flow cytometry analysis revealed progressive accumulation of cells in the sub-G1 phase, as well as an increase in the Annexin V-positive population of both cell lines. In TC71 cells, the sub-G1 fraction increased from 2.4% to 68.5%, and Annexin V-positive cells from 1.7% to 51.0%, by 96 h (*p* < 0.01; Supplementary Figure S7). In SK-ES-1 cells, the sub-G1 cell population increased from 3.4% to 35.1%, and that of Annexin V-positive cells from 1.9% to 51.6% (*p* < 0.01; Supplementary Figure S8).

Western blot analyses of cells treated with 1 μM AVN944 for 24 and 48 h revealed reduced levels of Cyclin D1, Cyclin E, and Bcl-2, accompanied by increased levels of Bax and cleaved PARP1, in both cell lines (Supplementary Figures S9 and S10). Notably, the strong induction of p53 observed at 5 μM was not detected at 1 μM, indicating that the magnitude of p53 activation is dose-dependent. Despite this attenuation, the overall pattern of cell cycle arrest and apoptotic signaling was consistent with that seen at higher concentrations.

Together, these data demonstrate that AVN944 induces cell cycle arrest and apoptosis of Ewing's sarcoma cells even at 1 μM, and that these effects are quantitatively weaker, but mechanistically identical, to those observed at 5 μM. This supports the view that AVN944 acts as a specific inhibitor of IMPDH2, rather than as a nonspecific cytotoxic agent.

### Inhibition of IMPDH2 by MPA recapitulates the apoptotic effects of AVN944

To confirm that the anti-tumor activity of AVN944 is attributable directly to inhibition of IMPDH2, we treated TC71 and SK-ES-1 cells with 1 μM MPA and analyzed the effects on the cell cycle and apoptosis over 96 h. In TC71 cells, the sub-G1 fraction increased gradually from 1.2% to 7.1%, and the percentage of Annexin V-positive cells increased from 0.9% to 13.7%, accompanied by a decrease in the percentage of viable cells (from 98.0% to 83.1%; *p* < 0.01; Supplementary Figure S11). A similar trend was observed for SK-ES-1 cells, with the sub-G1 fraction increasing from 1.0% to 11.2%, and the Annexin V-positive fraction from 2.0% to 16.9%, by 96 h (*p* < 0.01; Supplementary Figure S12). Although the apoptotic responses to MPA were weaker than those induced by AVN944 at 1 μM (Supplementary Figures S7 and S8), the patterns of cell cycle perturbation and apoptosis induction were remarkably similar. These results provide strong mechanistic evidence that IMPDH2 is the primary molecular target mediating the pro-apoptotic and anti-proliferative effects of AVN944 against Ewing's sarcoma cells.

### AVN944 inhibits growth of TC71 tumors in a xenograft model

**Figure 10:**
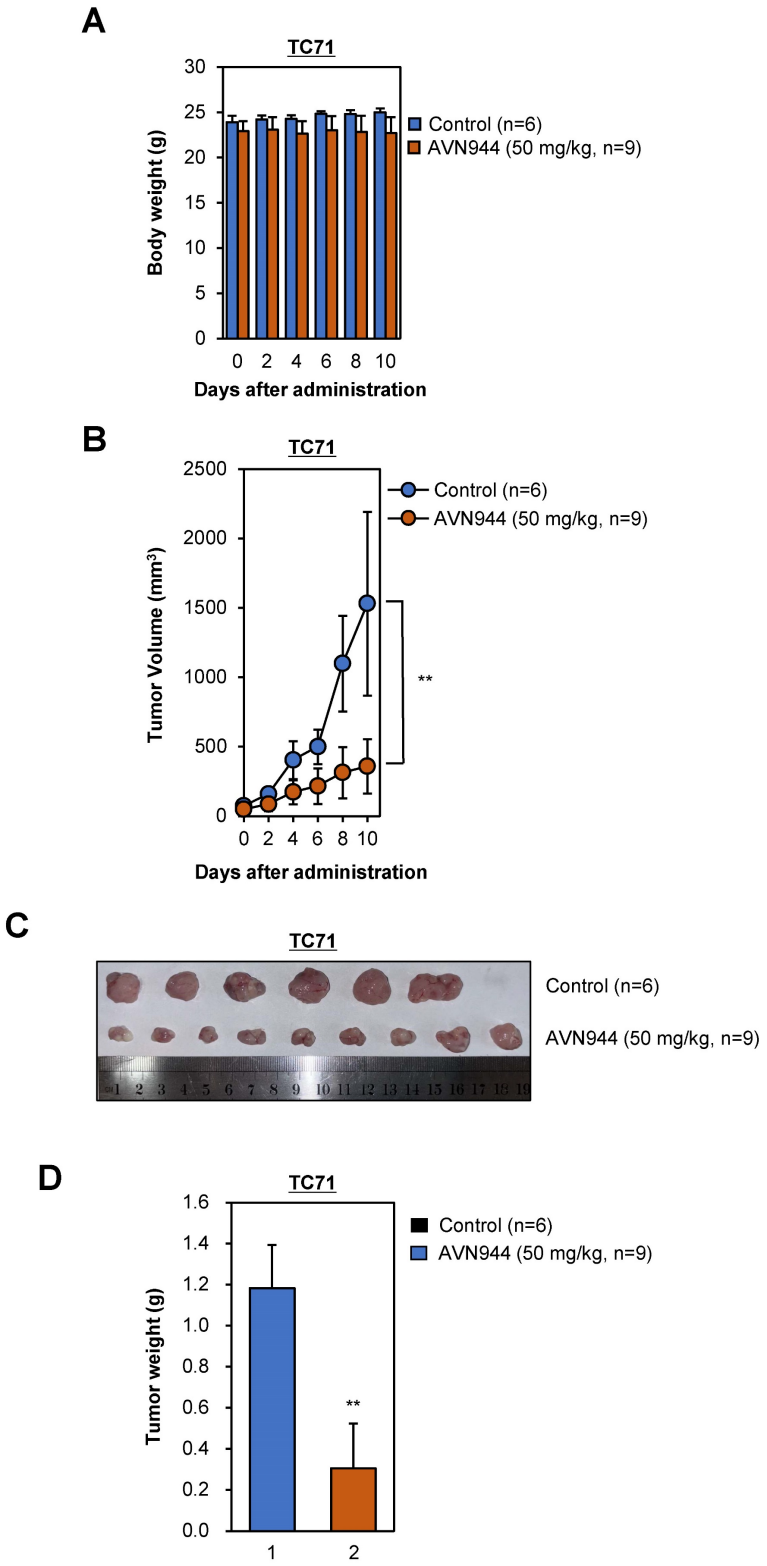
***In vivo* anti-tumor activity of AVN944 in a TC71 xenograft model**. **(A)** The body weight of mice treated with AVN944 (50 mg/kg) or control was monitored over 10 days. There was no significant difference in body weight changes between the AVN944-treated and the control groups, suggestive of no major systemic toxicity. Data are presented as the mean ± SD (control: n = 6; AVN944: n = 9). **(B)** Volume of tumors in TC71 xenograft-bearing mice treated with AVN944 (50 mg/kg, n = 9) compared with that of tumors in mice treated with the control (n = 6). Tumor growth in the AVN944-treated group was inhibited significantly, and tumor volume remained significantly smaller throughout the experiment (**p < 0.01). **(C)** Representative images of tumors excised from control (n = 6) and AVN944-treated mice (n = 9) at the end of the experiment. Tumors from AVN944-treated mice were markedly smaller than those from control mice, providing visual confirmation of the significant inhibition of tumor growth observed in Figure 10B. Each tumor was measured and aligned for comparison, demonstrating a clear reduction in tumor mass after AVN944 treatment**. (D)** AVN944-induced reductions in tumor weight in TC71 xenografts. Tumor weight was measured post-excision; AVN944 treatment led to a significant reduction in tumor mass when compared with the control (**p < 0.01). Data are presented as the mean ± SD (control: n = 6; AVN944: n = 9).

To evaluate the effects of AVN944 on tumor growth *in vivo*, we performed a xenograft experiment using TC71 Ewing's sarcoma cells. Nude mice were injected subcutaneously with TC71 cells and tumors were allowed to grow until they reached a volume of 15-25 mm^3^. Subsequently, mice were randomized into two groups: one group received AVN944 at a dose of 50 mg/kg for 10 days, whereas the other received vehicle control for 10 days. Tumor volumes and body weight were measured periodically, and at the end of the study, the tumors were excised, weighed, and photographed. Throughout the 10-day treatment period, body weight measurements served as a preliminary indicator of systemic toxicity associated with AVN944 administration. Statistical analysis indicated no significant differences in body weight between the control and treated groups (Figure 10A). These findings suggest that AVN944 does not induce acute toxicity at a dose of 50 mg/kg.

The anti-tumor activity of AVN944 was quantitatively assessed by monitoring tumor volumes in nude mice implanted subcutaneously with TC71 cells. Initial tumor volumes were standardized between 15-25 mm² before random division into two groups. Over the course of the experiment, the control group exhibited progressive tumor growth. By contrast, the AVN944-treated group showed significant inhibition of tumor growth, which was particularly noticeable from Day 4 onwards (Figure 10B). Statistical analysis confirmed that the differences in tumor volumes were significant, thereby validating the efficacy of AVN944 in suppressing growth of TC71 Ewing's sarcoma (**p < 0.01).

At the end of the experiment, tumors were excised from euthanized mice. Visual assessment corroborated the quantitative data; tumors in the AVN944-treated group were markedly smaller than those in the control group (Figure 10C). This visual difference was confirmed by weighing the tumors, which revealed that the average tumor weight in the AVN944 group was significantly lower than that in the control group (**p < 0.01, Figure 10D). These weight measurements are consistent with the volumetric data, and provide additional evidence supporting the anti-tumor efficacy of AVN944. Taken together, the data presented here demonstrate that AVN944 effectively inhibits growth of TC71 Ewing's sarcoma tumors in a xenograft model without exerting harmful systemic effects.

### AVN944 shows potent anti-tumor activity against SK-ES-1 Ewing's sarcoma xenografts

**Figure 11:**
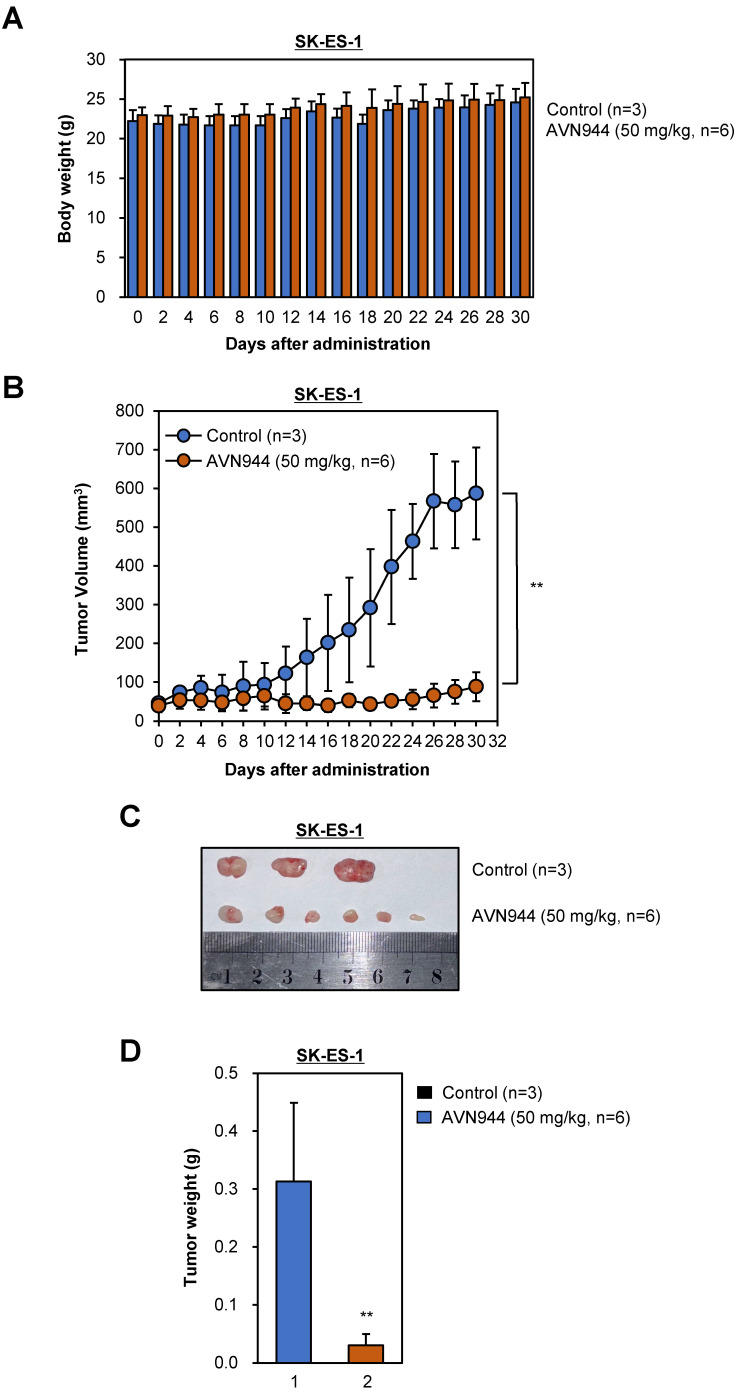
** Therapeutic potential of AVN944 in the SK-ES-1 Ewing's Sarcoma cell line. (A)** Longitudinal tracking of the body weight of SK-ES-1 xenograft-bearing mice. This panel depicts the body weight of mice over a 30-day period post-treatment with either the control (n=3) or AVN944 (50 mg/kg, n=6). The data illustrate that both groups maintained similar body weights throughout the experiment, suggesting that AVN944 does not exert significant systemic toxicity at the dose used. **(B)** AVN944 inhibits growth of tumors in SK-ES-1 xenograft mice. The graph illustrates dynamic changes in tumor volume in mice treated with AVN944 (n=6) or the control (n=3). The AVN944-treated group exhibited a significant reduction in tumor growth over time, demonstrating the compound's potent anti-tumor efficacy. The error bars indicate the SD, providing a clear visual of the statistical significance of tumor suppression (**p < 0.01). **(C)** Representative tumors from SK-ES-1 xenograft mice. Photograph of tumors excised at the end of the treatment period, providing a visual demonstration of the effects of AVN944 on tumor size. Smaller tumors in the AVN944 group (n=6) than in the control group (n = 3) visually corroborate the quantitative data regarding tumor suppression, highlighting the drug's effectiveness. **(D)** Comparison of tumor weights post-treatment with AVN944. The bar graph shows the final weight of tumors excised from the control (n=3) and AVN944-treated groups (n = 6), with the tumor mass in the AVN944 group being reduced significantly. This figure provides quantitative confirmation of the anti-tumor effects of AVN944 observed during the tumor volume and visual assessments, further supported by statistical analysis. The bar graphs display the mean weight ± SD, and highlight the statistical significance of the data, emphasizing the substantial impact of AVN944 on tumor size (**p<0.01).

We also evaluated the efficacy of AVN944 in an SK-ES-1 Ewing's sarcoma xenograft model. Over a 30-day period, we assessed its impact on SK-ES-1 cell tumor growth, and explored potential systemic toxicity by monitoring body weight. Throughout the experimental period (a 30-day period), neither the AVN944-treated group nor the control group displayed significant differences in body weight (Figure 11A), indicating a lack of acute or severe systemic toxicity at the dosage used. The therapeutic efficacy of AVN944 in an SK-ES-1 Ewing's sarcoma xenograft model was pronounced, with a significant reduction in tumor growth observed in the treated group compared with the control (Figure 11B).

The reduction in tumor volume was rapid, and was sustained throughout the treatment period, suggesting that AVN944 effectively inhibits cellular pathways critical for growth of SK-ES-1 cells. Post-mortem analyses included photographic documentation and weighing of SK-ES-1 tumors (Figures 11C and D). The visual and weight comparisons at the end of the study provided clear, tangible evidence of the impact of AVN944, with treated mice exhibiting notably smaller and lighter SK-ES-1 cell tumors than the control group. These results demonstrate that AVN944 exerts potent anti-tumor activity and minimal toxicity against Ewing's sarcoma cells, and that AVN944 is a promising candidate for further preclinical development, with potential application as a treatment for Ewing's sarcoma.

## Discussion

**Figure 12:**
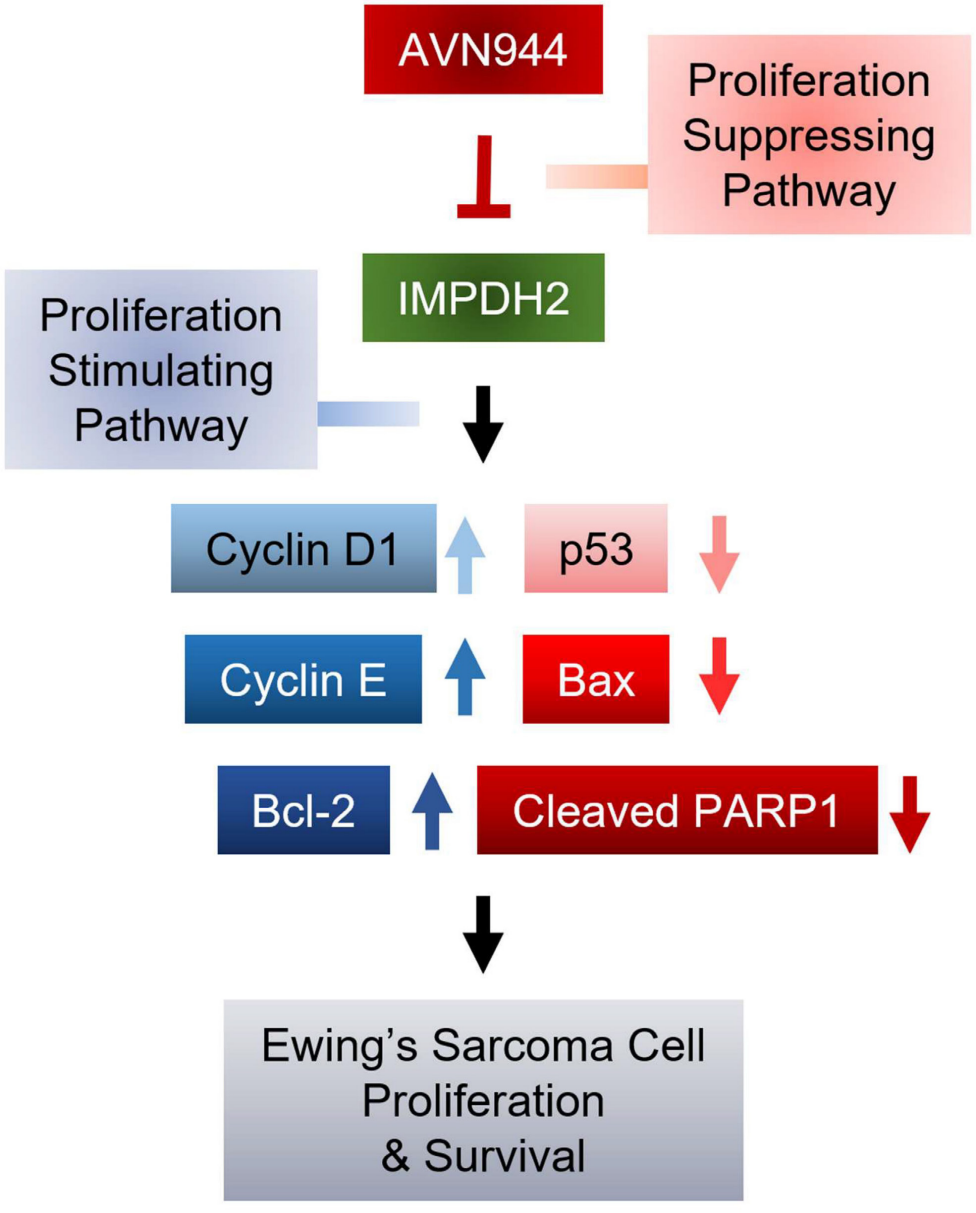
** Mechanism by which AVN944 in inhibits proliferation and survival of Ewing's Sarcoma cells.** Schematic diagram illustrating the mechanisms by which AVN944, an IMPDH2 inhibitor, inhibits growth of Ewing's sarcoma. AVN944 targets proliferation stimulating pathways by inhibiting IMPDH2, leading to a reduction in expression of several critical cellular growth regulators such as Cyclin D and Cyclin E, while also reducing expression of Bcl-2, an anti-apoptotic protein. Concurrently, AVN944 increases expression of pro-apoptotic factors such as Bax, and facilitates cleavage of PARP1, culminating in reduced tumor cell proliferation and increased apoptosis. This comprehensive depiction facilitates understanding of how AVN944 disrupts both cell cycle progression and survival pathways, offering insight into its potential as a therapeutic agent.

The current study evaluated the mechanistic efficacy of AVN944 as a modulator of cellular pathways involved in progression of Ewing's sarcoma. By targeting specific molecular pathways, AVN944 offers a strategic approach to mitigating the rapid proliferation of Ewing's sarcoma cells. Treatment with AVN944 resulted in a dose-dependent reduction in cell viability, corroborating its role as a potent inhibitor of cellular proliferation. The decline in viability was particularly notable at higher concentrations, highlighting the drug's cytotoxic potential at elevated doses. Moreover, AVN944 treatment led to significant changes in expression of key regulators of the cell cycle and apoptosis. This suggests that AVN944 effectively halts the cell cycle, preventing proliferation of Ewing's sarcoma cells. Simultaneously, increases in expression of p53 and Bax, and a decrease in expression of Bcl-2 were noted, indicating activation of apoptotic pathways. This was further evidenced by a critical event in the execution phase of apoptosis: increased cleavage of PARP1. These molecular alterations reveal that AVN944 not only inhibits cell division, but also induces death of Ewing's sarcoma cells, making it a dual-action therapeutic agent (Figure 12).

We focused on expression of IMPDH2 in Ewing's sarcoma cell lines, and explored its potential as a therapeutic target in Ewing's sarcoma. Our findings from the TCGA cohort reveal that elevated expression of IMPDH2 correlates with a trend toward reduced OS in patients with sarcoma. Although Kaplan-Meier analysis did not reach statistical significance (*p* = 0.061), the hazard ratio of 1.5 suggests that high expression of IMPDH2 could be a risk factor for poorer outcomes. These findings are consistent with those of previous studies linking high IMPDH2 expression to adverse prognoses for other malignancies, including colorectal 25, 26 and breast cancers 15, in which IMPDH2 is thought to promote tumor cell proliferation and resistance to apoptosis. Differential expression analyses further highlighted marked overexpression of IMPDH2 in sarcoma tissues compared with normal tissues. This overexpression suggests a potential role for IMPDH2 in sarcoma pathogenesis, potentially by supporting the increased nucleotide synthesis required for rapid cell proliferation of malignant cells. Because IMPDH2 is a key enzyme in the *de novo* guanine nucleotide synthesis pathway 13, 24, its upregulation could provide a metabolic advantage to cancer cells, thereby facilitating growth and survival. Western blot analysis confirmed high expression of the IMPDH2 protein expression in three representative Ewing's sarcoma cell lines (RD-ES, SK-ES-1, and TC71), and provided additional validation of the TCGA data at the protein level. The presence of IMPDH2 in these sarcoma cell lines supports its relevance to this cancer type, and underscores its potential as a therapeutic target.

While our data suggest a trend toward decreased survival of patients with elevated expression of IMPDH2, we acknowledge that the reported *p*-value (*p* = 0.061) does not meet conventional thresholds for statistical significance. This limitation may reflect the relatively small sample size of TCGA sarcoma cohort, as well as intrinsic heterogeneity across sarcoma subtypes 3, 27, 28, which could affect the generalizability of our findings. Given that sarcomas are a rare and diverse group of malignancies with distinct molecular characteristics, future studies with larger and more homogeneous patient cohorts are necessary to clarify the prognostic value of IMPDH2 expression in sarcoma, and to determine its variability across different subtypes. Although the current analysis does not establish IMPDH2 as a statistically significant prognostic marker, its potential utility as a biomarker for identifying patients who may benefit from specific therapies warrants further investigation. For example, IMPDH2 inhibitors could be explored as adjunct therapies for sarcoma treatment. Additional *in vitro* and *in vivo* studies are warranted to assess the effects of IMPDH2 inhibition on sarcoma cell viability and tumor growth, as well as to evaluate potential resistance mechanisms.

Importantly, the present study provides comprehensive evidence that the IMPDH2 inhibitor AVN944 exerts potent anti-proliferative and anti-clonogenic effects against two Ewing's sarcoma cell lines, TC71 and SK-ES-1. These results underscore the therapeutic potential of AVN944 as a targeted agent for treating Ewing's sarcoma; however, the tumor is still challenging to treat because of its aggressive nature and limited therapeutic options. In TC71 cells, AVN944 inhibited cell proliferation, reduced BrdU incorporation, and suppressed colony formation significantly and in a dose-dependent manner. Morphological changes, coupled with a pronounced decrease in cell density, suggest that AVN944 disrupts cellular homeostasis, likely by depleting intracellular pools of GTP. This finding aligns with previous studies of other cancer types such as prostate cancer cells and multiple myeloma cells, in which AVN944 induces cell cycle arrest and apoptosis by inhibiting IMPDH2 17, 19. Similarly, AVN944 exerted robust anti-proliferative effects against SK-ES-1 cells, with significant reductions in BrdU-positive cells and cell viability. The consistent inhibition of colony formation by both cell lines highlights the ability of the compound to target long-term self-renewal and survival capacity, both of which are critical for progression and/or relapse of Ewing's sarcoma.

The findings presented herein demonstrate that AVN944 exerts potent pro-apoptotic effects on TC71 and SK-ES-1 cells, as evidenced by the time-dependent increase in sub-G1 and Annexin V-positive cell populations, coupled with a marked reduction in cell viability. These observations suggest that AVN944 effectively induces apoptosis of Ewing's sarcoma cells, corroborating its potential as a therapeutic agent for this malignancy. Previous studies show that inhibiting IMPDH2 disrupts nucleotide synthesis, leading to cell cycle arrest and apoptosis, particularly in rapidly proliferating cancer cells 17, 29, 30. Our data align with these findings; AVN944 treatment resulted in significant accumulation of cells in the sub-G1 phase, indicative of DNA fragmentation and apoptosis. The progressive increase in sub-G1 populations from 48 to 96 h post-treatment highlights the time-dependent nature of apoptosis induction in TC71 and SK-ES-1 cells. To summarize, the data underscore the therapeutic potential of AVN944 as an apoptosis-inducing agent for Ewing's sarcoma. The time-dependent apoptotic effects observed in TC71 and SK-ES-1 cells provide a strong rationale for further preclinical investigations. Future studies should focus on evaluating the *in vivo* efficacy of AVN944, and its potential for use in combination with other chemotherapeutic agents to enhance treatment outcomes of patients with Ewing's sarcoma.

It is remarkable that in both TC71 and SK-ES-1 cells, AVN944 induced robust, time-dependent upregulation of p53, coupled with significant downregulation of Cyclin D1 and Cyclin E. Suppression of Cyclin D1 and Cyclin E further supports the hypothesis that AVN944 exerts anti-proliferative effects through cell cycle arrest. Comparable findings have been reported by studies utilizing other metabolic inhibitors such as ribonucleotide reductase inhibitors, in which induction of p53 and suppression of cyclins effectively halted cell cycle progression 31-33. The data also underscore the potent pro-apoptotic effects of AVN944, as evidenced by the increased Bax/Bcl-2 ratio and robust cleavage of PARP1 in both cell lines. Upregulation of Bax, alongside concomitant downregulation of Bcl-2, align with activation of intrinsic apoptotic pathways, and are consistent with observations made in other studies involving purine metabolism inhibitors 34-36. There was a marked increase in the amount of cleaved PARP1, a hallmark of caspase-mediated apoptosis, following AVN944 treatment, reinforcing its role in facilitating DNA fragmentation and apoptotic cell death. Comparative analysis of our findings highlights the potential of AVN944 as a dual-acting agent that targets both cell cycle progression and apoptotic pathways. Comprehensive genomic and proteomic profiling of Ewing's sarcoma cell lines treated with AVN944 could uncover biomarkers that predict responses to treatment, thereby enhancing patient stratification strategies.

It is worth noting that the efficacy of AVN944 as an inhibitor of TC71 and SK-ES-1 Ewing's sarcoma xenograft growth supports its value as a promising therapeutic candidate that merits further exploration within the context of current oncological research. Both of the xenograft models utilized in these studies underscore the complex interaction between therapeutic agents and tumor pathophysiology, echoing the necessity for robust preclinical models to evaluate anticancer drugs effectively. Furthermore, the findings from these models provide critical insight into the dynamics of tumor growth and responses to treatment. Similarly, our study with AVN944 shows that it inhibits tumor growth significantly, without inducing noticeable systemic toxicity, supporting the current understanding that an effective treatment for Ewing's sarcoma must balance potency with a manageable safety profile. Moreover, the lack of systemic toxicity exerted by AVN944, as evidenced by maintenance of body weight across treatment periods, aligns with other contemporary studies emphasizing the need for therapies that do not exacerbate the patient's condition by triggering adverse side effects. The reductions in tumor volume without weight loss further supports the role of AVN944 as a potent inhibitor of tumor growth, a significant marker of its therapeutic potential.

While the results of this study provide promising insights into the potential of AVN944 as a therapeutic agent for Ewing's sarcoma, several limitations should be acknowledged to fully contextualize the scope and applicability of our findings. This study primarily investigated the effects of AVN944 as a single agent, demonstrating significant anti-tumor activity in preclinical models; however, the complex biology of aggressive cancers such as Ewing's sarcoma often necessitates combination therapy to overcome adaptive resistance mechanisms and to achieve more durable clinical responses. Given that multi-agent chemotherapy remains the standard of care for Ewing's sarcoma, it is essential to consider whether AVN944 would be best positioned as a monotherapy, or used in combination with established chemotherapeutic agents such as doxorubicin or vincristine, or with molecularly targeted therapies. Investigating combinatorial strategies incorporating AVN944 may increase therapeutic efficacy while potentially mitigating resistance and toxicity associated with high-dose single-agent treatments. Future studies should focus on identifying synergistic drug combinations, as well as determining optimal sequencing and dosing strategies to maximize therapeutic outcomes while minimizing adverse effects, thereby facilitating translation of AVN944 into clinically effective regimens for patients with Ewing's sarcoma.

While this study utilized a TCGA sarcoma cohort to investigate gene expression patterns associated with patient outcomes, it is important to acknowledge the limitations inherent to this dataset. Although the TCGA sarcoma cohort contains a substantial number of patient-derived tumor samples (n = 262), it includes only two normal tissue samples due to the challenges of obtaining matched normal soft tissue controls. This limitation constrained our comparative analysis between sarcoma and normal tissues, and we were unable to increase the number of normal samples within the scope of the current dataset. We have taken care to avoid overinterpretation of the comparative findings in Figure 1 by recognizing that the small number of normal controls limits the statistical power and generalizability of these comparisons. Future studies should incorporate a larger number of normal tissue samples from independent cohorts to validate and extend these findings, thereby enabling a more robust assessment of differences in gene expression between sarcoma and normal tissues. Despite this limitation, and given the comprehensive genomic and transcriptomic profiling of a diverse range of sarcoma subtypes within this cohort, the analysis of the TCGA dataset remains valuable for identifying potential biomarkers and therapeutic targets in sarcoma. This approach can guide further mechanistic studies and validation using independent datasets and additional clinical samples to increase the translational relevance of these findings.

In conclusion, the data presented herein not only support the continued development of AVN944 as a potential treatment for Ewing's sarcoma, but also contribute to the broader discourse regarding the necessity of using clinically-relevant models for preclinical testing. This ensures that therapeutic agents are not only effective at reducing tumor burden, but also maintain a profile that is tolerable for patients, thereby improving the overall prognosis and quality of life for those affected by this challenging malignancy. Further studies should focus on elucidating the detailed mechanisms through which AVN944 exerts its effects, and on exploring its potential applications across different Ewing's sarcoma models. This will pave the way for transitioning from preclinical trials to clinical applications, in which the real-world efficacy and safety of AVN944 in patients with Ewing's sarcoma can be evaluated thoroughly.

## Supplementary Material

Supplementary materials and methods, figures.
