# Supplementary material for: AVN944 Elicits Apoptotic Responses and Impedes Tumorigenic Potential in Ewing's Sarcoma Cells
Source: Int J Biol Sci. 2026 Jan 1;22(2):529–52. doi: 10.7150/ijbs.116651 (PMC12780844; doi:10.7150/ijbs.116651)

AVN944 Elicits Apoptotic Responses and Impedes Tumorigenic Potential in Ewing's Sarcoma Cells

Hanah Lim, Seonock Lee, Gamin Kim, Eun Joo Lee, and Jungho Kim

Supplementary Materials

Supplementary Materials and Methods

Cell culture of normal human cell lines (MRC-5, MSC, and HDF)

Normal human cell lines were cultured under standard conditions (37°C/5% CO_2) in a humidified incubator (Thermo Fisher Scientific). MRC-5 (human fetal lung fibroblast; ATCC) cells were maintained in Minimum Essential Medium (MEM) supplemented with 10% heat-inactivated FBS, GlutaMAX, and 1% penicillin-streptomycin. Bone marrow-derived mesenchymal stem cells (MSCs; ATCC) were cultured using the Mesenchymal Stem Cell Growth Kit (ATCC), in medium supplemented with 7% heat-inactivated FBS (Sigma-Aldrich), recombinant human IGF-1 (15 ng/mL), recombinant human FGF-b (125 ng/mL), and L-alanyl-L-glutamine (2.4 mM). Human dermal fibroblasts (HDFs; ATCC) were cultured in Dulbecco's Modified Eagle's Medium (DMEM) containing 10% heat-inactivated FBS, GlutaMAX, and 1% penicillin-streptomycin. All cells were maintained in a humidified incubator at 37°C/5% CO_2 and subcultured when they reached 70–80% confluence.

Evaluation of additional IMPDH2 inhibitors using Ewing's sarcoma cell lines

To confirm that the anti-proliferative and anti-clonogenic effects of AVN944 result from inhibition of IMPDH2 rather than compound-specific off-target activity, two structurally and mechanistically distinct IMPDH2 inhibitors, mycophenolic acid (MPA) and sappanone A (SA), were tested against TC71 and SK-ES-1 Ewing's sarcoma cells. MPA and SA were dissolved in DMSO and diluted in culture medium immediately before use.

Cell growth, colony-formation, cell cycle, apoptosis, and immunoblot analyses following treatment with 1 μM AVN944

Treatment with 1 μM AVN944 was performed as described for 5 μM AVN944 (see the Materials and Methods section of the main text), unless specified otherwise. Briefly, TC71 and SK-ES-1 Ewing's sarcoma cells were cultured under standard conditions and then treated with 1 μM AVN944 or vehicle control (0.1% DMSO) for the indicated times (0–96 h). All experiments were repeated independently three times (n=3), and data presented as the mean ± SD.

Supplementary Figure Legend

Supplementary Figure S1. Kaplan–Meier survival analyses of GTP biosynthesis-related enzymes in the TCGA sarcoma cohort. Kaplan–Meier curves comparing overall survival of the groups showing high (red) and low (blue) expression of six enzymes involved in GTP biosynthesis: **(A)** GMPR, **(B)** GMPR2, **(C)** IMPDH1, **(D)** GUK1, **(E)** NME1, and **(F)** GMPS. Patients were stratified using median TPM expression as the cutoff. Each plot includes hazard ratios, log-rank *p* values, and dotted lines representing 95% confidence intervals. Although several comparisons did not reach statistical significance, most of the high expression groups showed a trend toward decreased survival, indicating that elevated activity of enzymes within the GTP biosynthesis pathway may contribute to a poor prognosis for those with sarcoma.

Supplementary Figure S2. Effects of MPA on TC71 cells. (A) Representative phase-contrast images showing time-dependent reduction in cell density over 5 days post-treatment with 1 μM MPA. **(B)** Growth curves demonstrate significant suppression of cell proliferation compared with vehicle-treated controls (**p < 0.01). **(C)** Colony-formation assay showing dose-dependent reduction in colony numbers (0–1 μM MPA). **(D)** Quantification of colony numbers, expressed as percentage of the control; colony formation was inhibited significantly at ≥ 0.125 μM (**p < 0.01). Data are presented as the mean ± SD of three independent experiments (n=3). All scale bars = 100 μm.

Supplementary Figure S3. Effects of SA on TC71 cells. (A) Representative images show reduced cell density after SA treatment (1 μM) for 5 days. **(B)** Growth curve demonstrating marked inhibition of cell proliferation (**p < 0.01). **(C)** Colony-formation assay showing a strong dose-dependent decline in colony number after SA treatment (0–1 μM). **(D)** Quantitative analysis revealing almost complete suppression of colony formation at ≥ 0.25 μM SA (*p < 0.05, **p < 0.01). Data are presented as the mean ± SD of three independent experiments (n=3). All scale bars = 100 μm.

Supplementary Figure S4. Effects of MPA on SK-ES-1 cells. (A) Representative phase-contrast images showing time-dependent reduction in cell density over 5 days post-treatment with 1 μM MPA. **(B)** Growth curves demonstrate significant suppression of cell proliferation compared with vehicle-treated controls (**p < 0.01). **(C)** Colony-formation assay showing dose-dependent reduction in colony numbers (0–1 μM MPA). **(D)** Quantification of colony numbers, expressed as percentage of the control; colony formation was inhibited significantly at ≥ 0.125 μM (**p < 0.01). Data are presented as the mean ± SD of three independent experiments (n=3). All scale bars = 100 μm.

Supplementary Figure S5. Effects of SA on SK-ES-1 cells. (A) Representative phase-contrast images showing time-dependent reduction in cell density over 5 days post-treatment with 1 μM SA. **(B)** Growth curves demonstrate significant suppression of cell proliferation compared with vehicle-treated controls (**p < 0.01). **(C)** Colony-formation assay showing dose-dependent reduction in colony numbers (0–1 μM SA). **(D)** Quantification of colony numbers, expressed as percentage of the control; colony formation was inhibited at ≥ 1 μM (**p < 0.01). Data are presented as the mean ± SD of three independent experiments (n=3). All scale bars = 100 μm.

Supplementary Figure S6. AVN944 suppresses proliferation of Ewing's sarcoma cells to a greater extent than normal human cells. (A, B) Ewing's sarcoma cell lines TC71 and SK-ES-1 exhibited profound growth inhibition, with remaining cell numbers equivalent to 0.2%

and 2.3% of the respective controls. **(C–E)** By contrast, the remaining normal human cells (MRC-5 fibroblasts, mesenchymal stem cells (MSCs), and human dermal fibroblasts (HDFs)) equated to 34.0%, 10.8%, and 9.4% of the control cell numbers after identical treatment. Representative phase-contrast images and quantification of relative cell numbers after 5 days of treatment with 1 μM AVN944. All data are expressed as the mean ± SD (n=3); $^{**}p < 0.01$ *vs.* control. These results demonstrate that AVN944 exhibits markedly higher cytotoxicity toward Ewing's sarcoma cells than toward normal cells, indicating tumor-selective sensitivity.

Supplementary Figure S7. Effects of 1 μM AVN944 on cell-cycle distribution and apoptosis of TC71 cells. (A–E) Flow cytometry analysis of TC71 cells treated with 1 μM AVN944 for 0–96 h. Representative DNA-content histograms show time-dependent increases in the sub-G1 population, indicating apoptosis. Quantification of sub-G1 ratios **(B)** and Annexin V-positive cell numbers **(D)** demonstrates progressive cell death, whereas the viable fraction **(E)** declines accordingly ($^{**}p < 0.01$). Data are presented as the mean ± SD (n=3).

Supplementary Figure S8. Effects of 1 μM AVN944 on cell-cycle distribution and apoptosis in SK-ES-1 cells. (A–E) Flow cytometry analysis of SK-ES-1 cells treated with 1 μM AVN944 for 0–96 h. Sub-G1 ratios increased from 3.4% to 35.1% by 96 h, accompanied by a rise in Annexin V-positive cells and a drop in viable cells ($^{**}p < 0.01$). These results confirm that AVN944 induces apoptosis in a time- and dose-dependent manner. Data are presented as the mean ± SD (n=3).

Supplementary Figure S9. Expression of cell cycle– and apoptosis-related proteins by TC71 cells treated with 1 μM AVN944. Western blot analysis of p53, Cyclin D1, Cyclin E, Bax, Bcl-2, and PARP1 levels in TC71 cells treated with 1 μM AVN944 for 0, 24, and 48 h. Down-regulation of Cyclin D1/E and Bcl-2, together with up-regulation of Bax and cleaved PARP1, was observed. Induction of p53 was not detectable at this low dose.

Supplementary Figure S10. Expression of cell cycle– and apoptosis-related proteins in SK-ES-1 cells following treatment with 1 μM AVN944. Western blot analysis of p53, Cyclin D1, Cyclin E, Bax, Bcl-2, and PARP1 levels in SK-ES-1 cells treated with 1 μM AVN944 for 0, 24, and 48 h. Corresponding results in SK-ES-1 cells show a similar pattern of Cyclin D1/E suppression and Bax activation, with minimal changes in p53 levels. These findings indicate that the molecular responses to AVN944 are concentration-dependent, yet mechanistically consistent with those observed at 5 μM.

Supplementary Figure S11. MPA induces apoptosis and cell-cycle arrest in TC71 cells. (A–E) Flow cytometry analysis of TC71 cells treated with 1 μM MPA for 0–96 h. **(A)** Representative DNA-content histograms show a time-dependent increase in the sub-G1 population (1.2% → 7.1% by 96 h). **(B)** Quantified sub-G1 ratios confirm progressive accumulation of apoptotic cells ($^{**}p < 0.01$). **(C, D)** Annexin V/PI staining demonstrates an increase in Annexin V-positive cells from 0.9% to 13.7% ($^{**}p < 0.01$), with a corresponding decline in the percentage of viable cells **(E)**.

Supplementary Figure S12. MPA induces apoptosis and cell-cycle arrest in SK-ES-1 cells. **(A–E)** Flow cytometry analysis of SK-ES-1 cells treated with 1 μM MPA for 0–96 h. **(A)** Representative DNA-content histograms show a time-dependent increase in the sub-G1 population (1.0% → 11.2% by 96 h). **(B)** Quantified sub-G1 ratios confirm progressive accumulation of apoptotic cells (**$p < 0.01$). **(C, D)** Annexin V/PI staining demonstrates an increase in Annexin V-positive cells (from 2.0% to 16.9%; **$p < 0.01$), with a corresponding decline in the percentage of viable cells **(E)**.

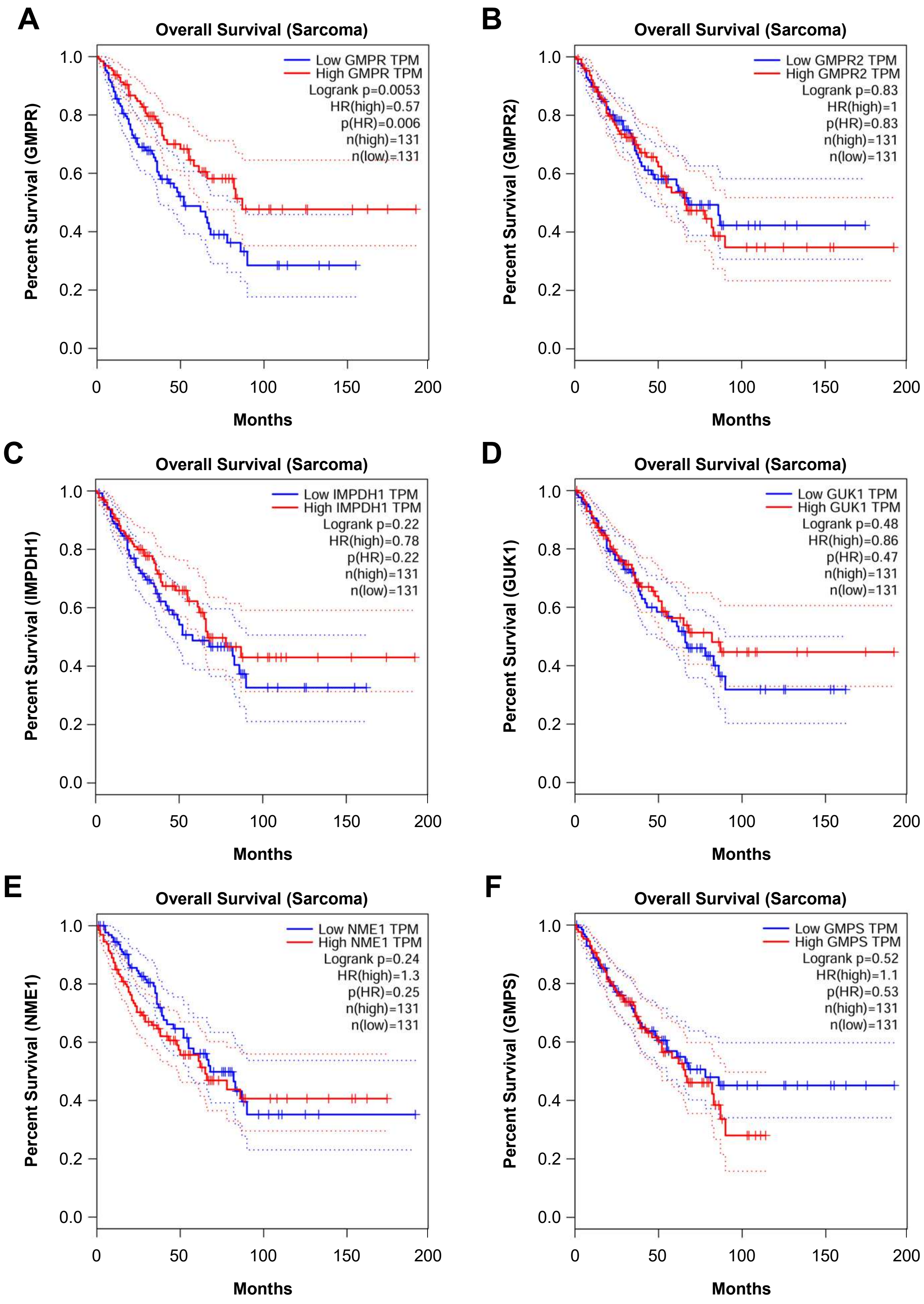
A
Overall Survival (Sarcoma)
Low GMPR TPM
High GMPR TPM
Logrank p=0.0053
HR(high)=0.57
p(HR)=0.006
n(high)=131
n(low)=131
Percent Survival (GMPR)
Months
B
Overall Survival (Sarcoma)
Low GMPR2 TPM
High GMPR2 TPM
Logrank p=0.83
HR(high)=1
p(HR)=0.83
n(high)=131
n(low)=131
Percent Survival (GMPR2)
Months
C
Overall Survival (Sarcoma)
Low IMPDH1 TPM
High IMPDH1 TPM
Logrank p=0.22
HR(high)=0.78
p(HR)=0.22
n(high)=131
n(low)=131
Percent Survival (IMPDH1)
Months
D
Overall Survival (Sarcoma)
Low GUK1 TPM
High GUK1 TPM
Logrank p=0.48
HR(high)=0.86
p(HR)=0.47
n(high)=131
n(low)=131
Percent Survival (GUK1)
Months
E
Overall Survival (Sarcoma)
Low NME1 TPM
High NME1 TPM
Logrank p=0.24
HR(high)=1.3
p(HR)=0.25
n(high)=131
n(low)=131
Percent Survival (NME1)
Months
F
Overall Survival (Sarcoma)
Low GMPS TPM
High GMPS TPM
Logrank p=0.52
HR(high)=1.1
p(HR)=0.53
n(high)=131
n(low)=131
Percent Survival (GMPS)
Months

Supplementary Figure S2

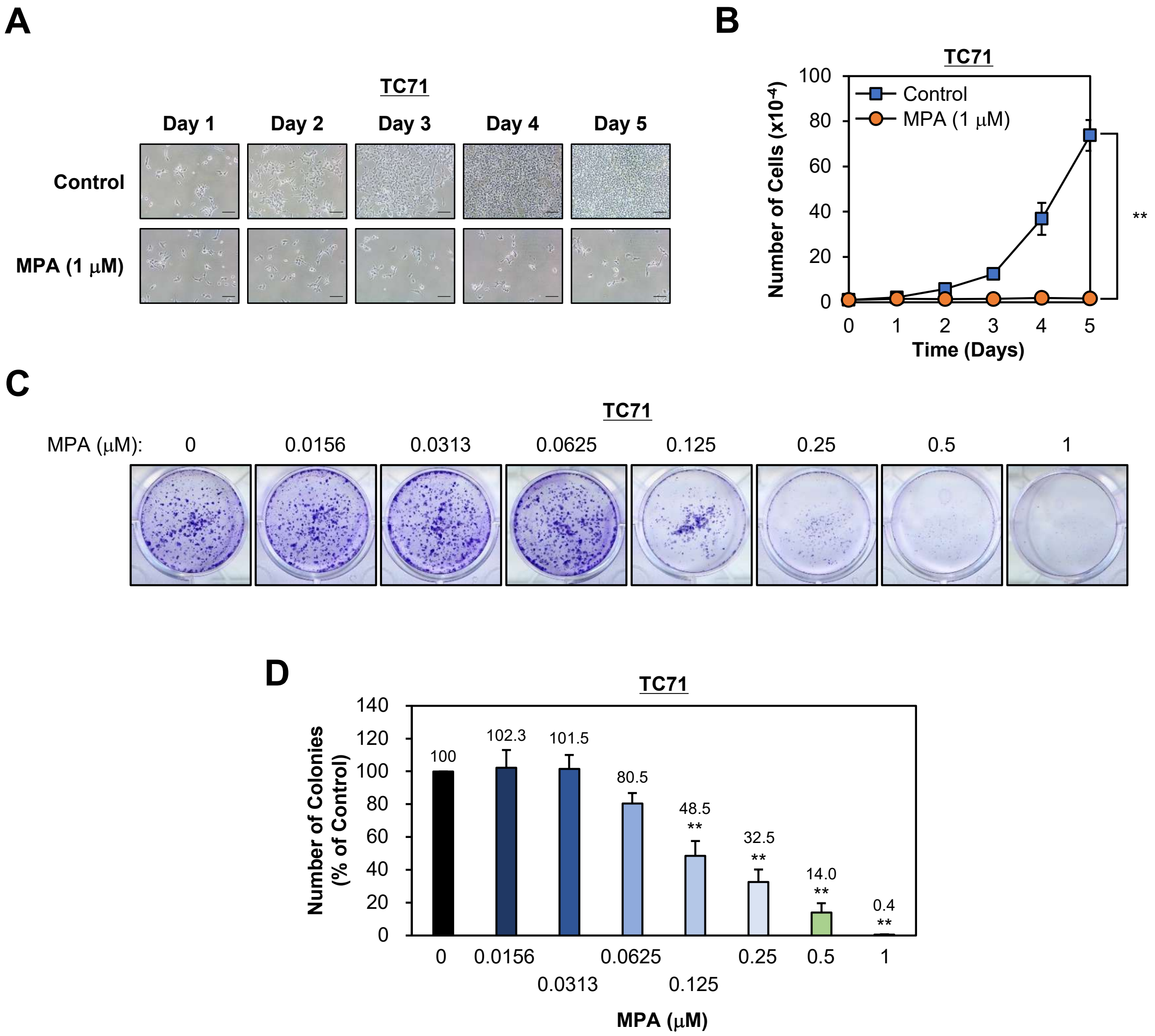

Supplementary Figure S3

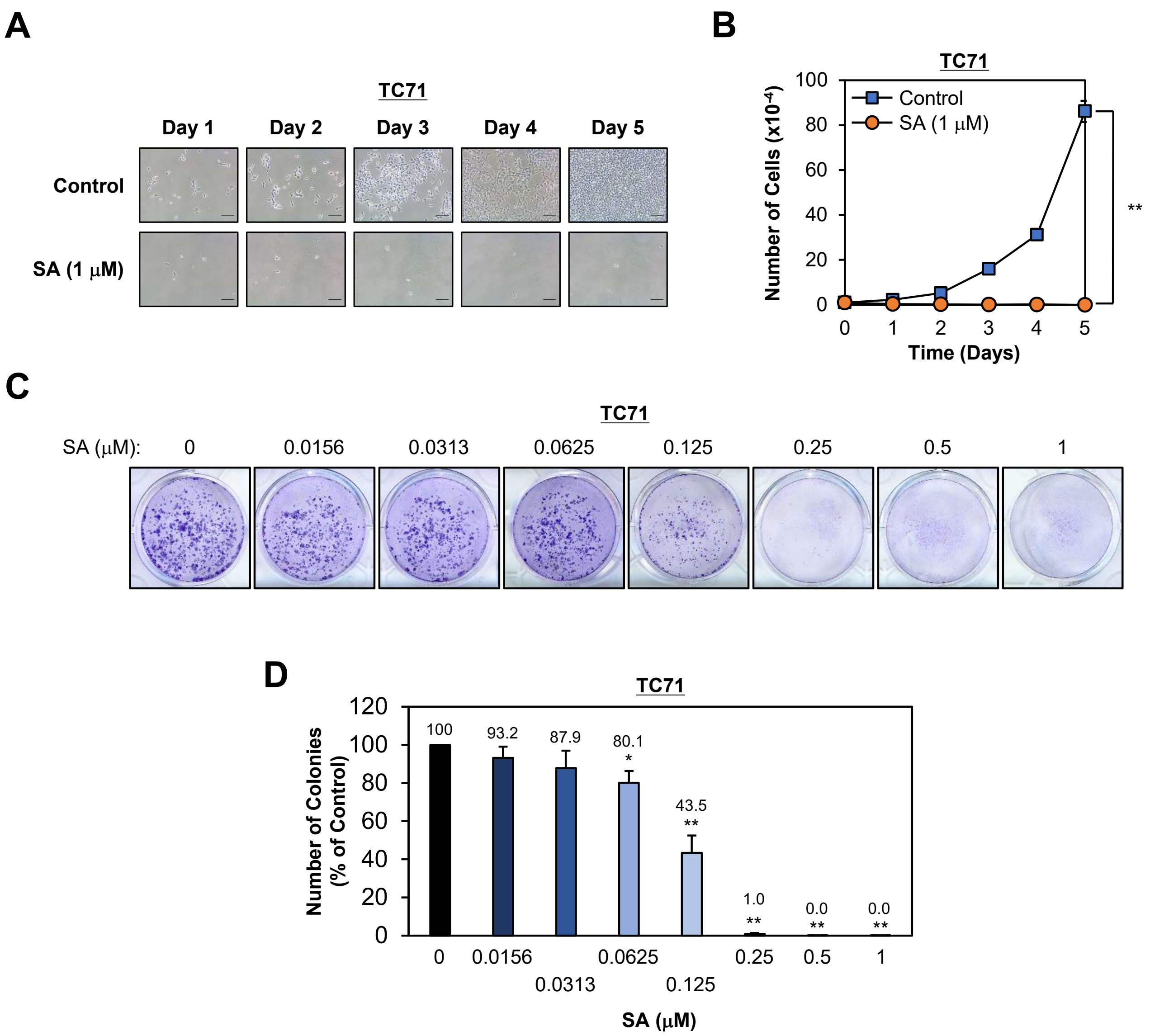

Supplementary Figure S4

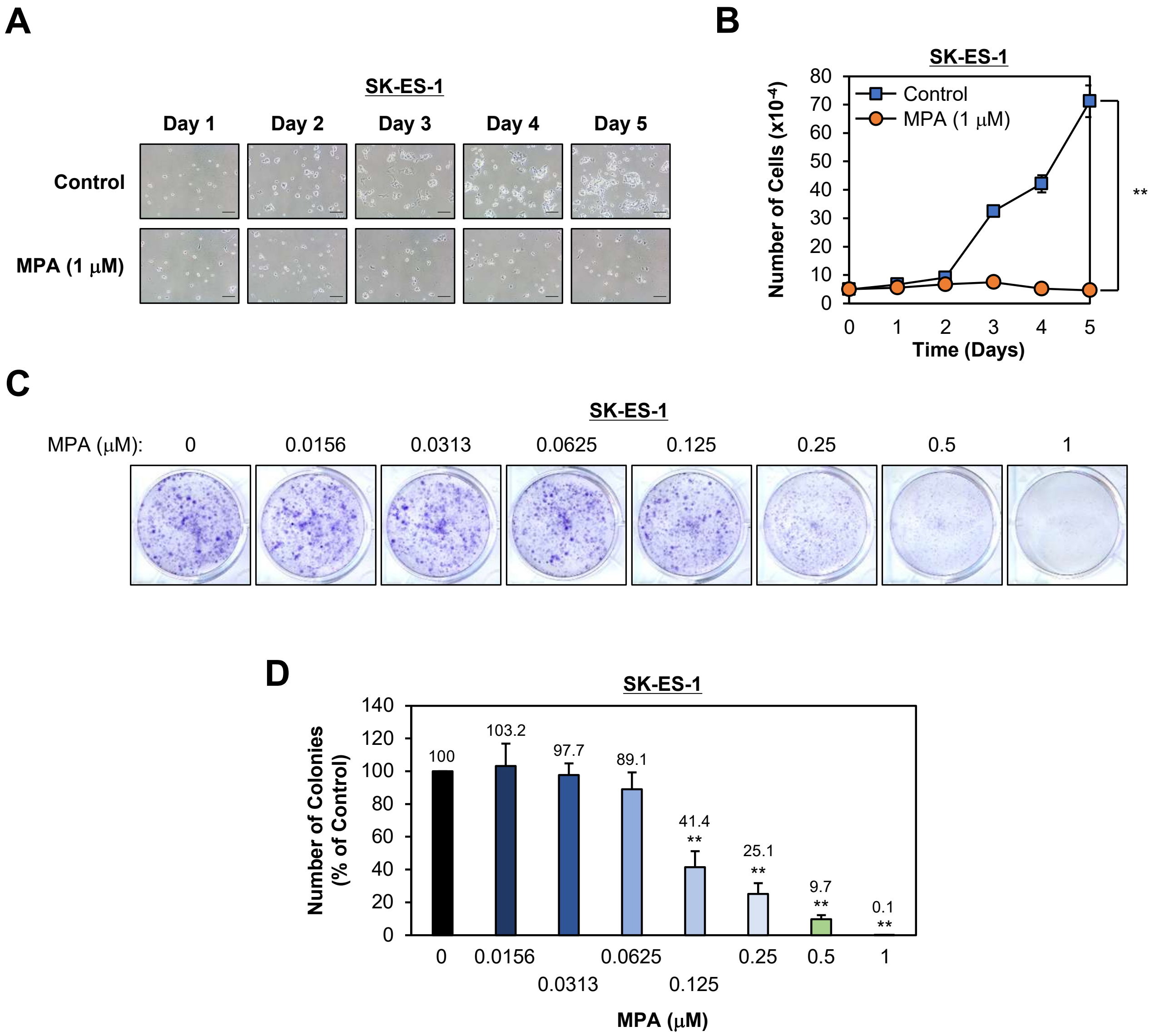

Supplementary Figure S5

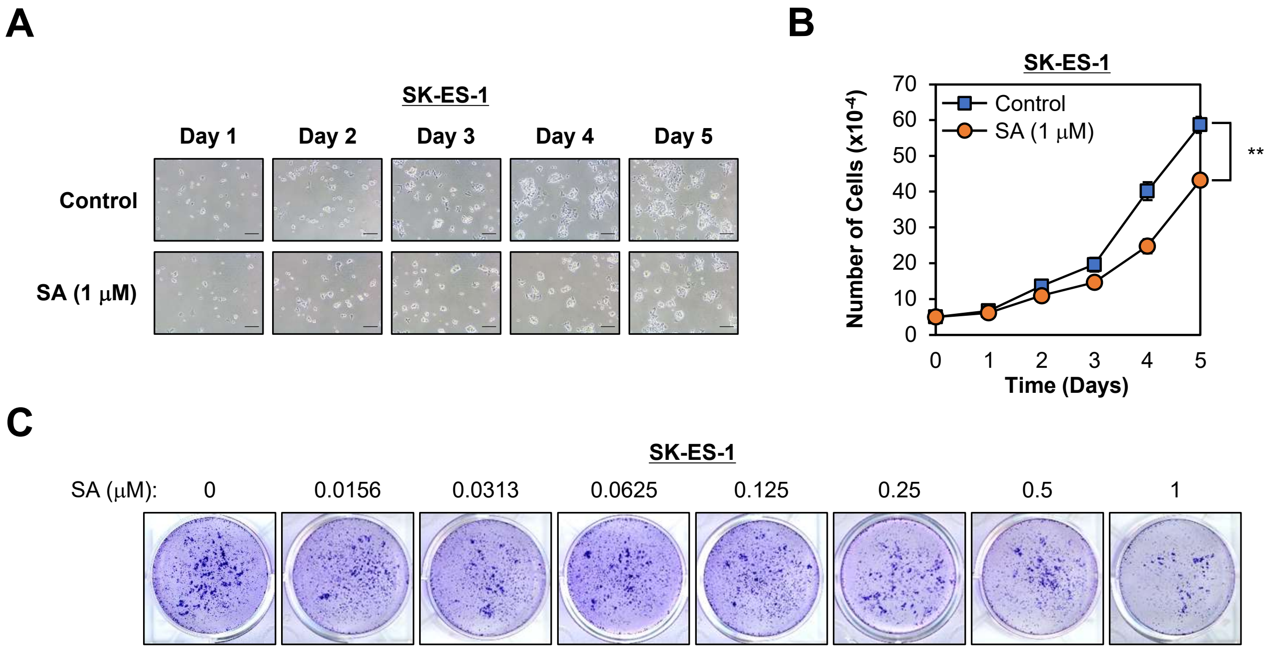

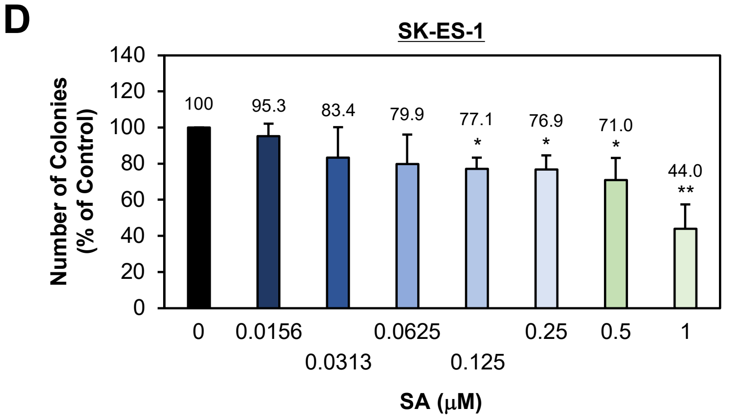

Supplementary Figure S6

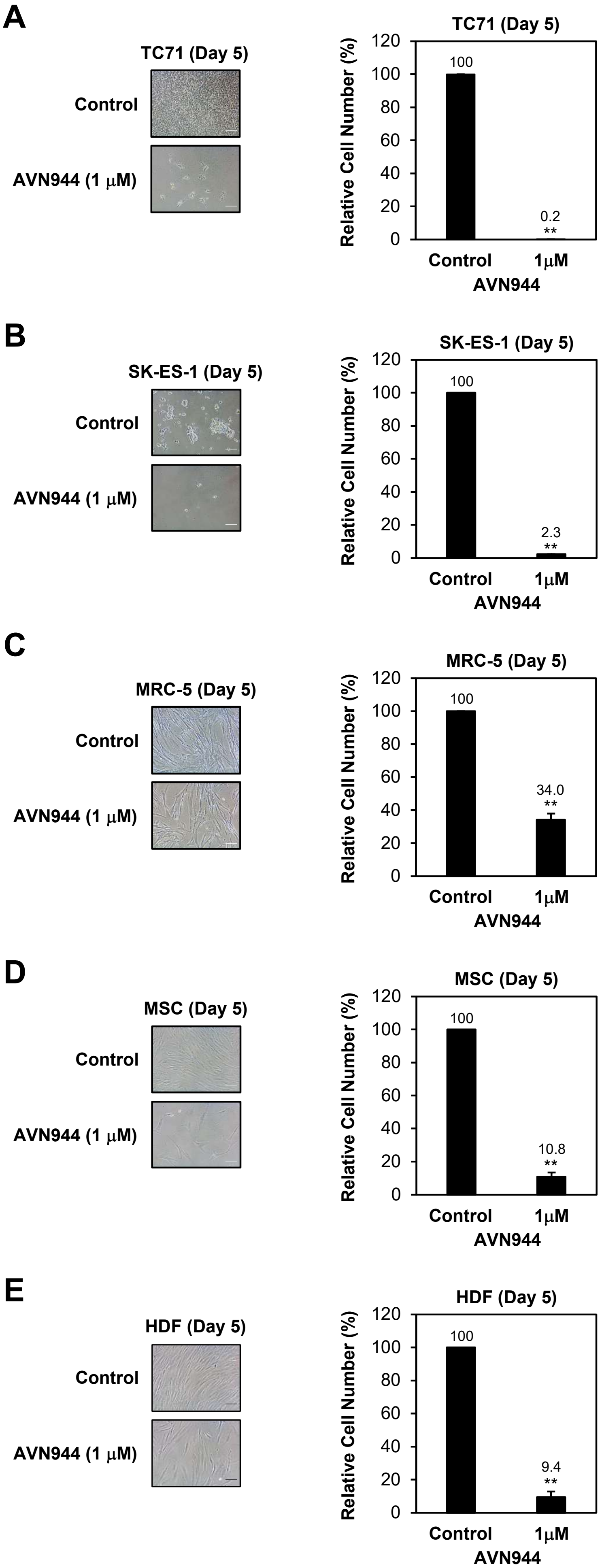

Supplementary Figure S7

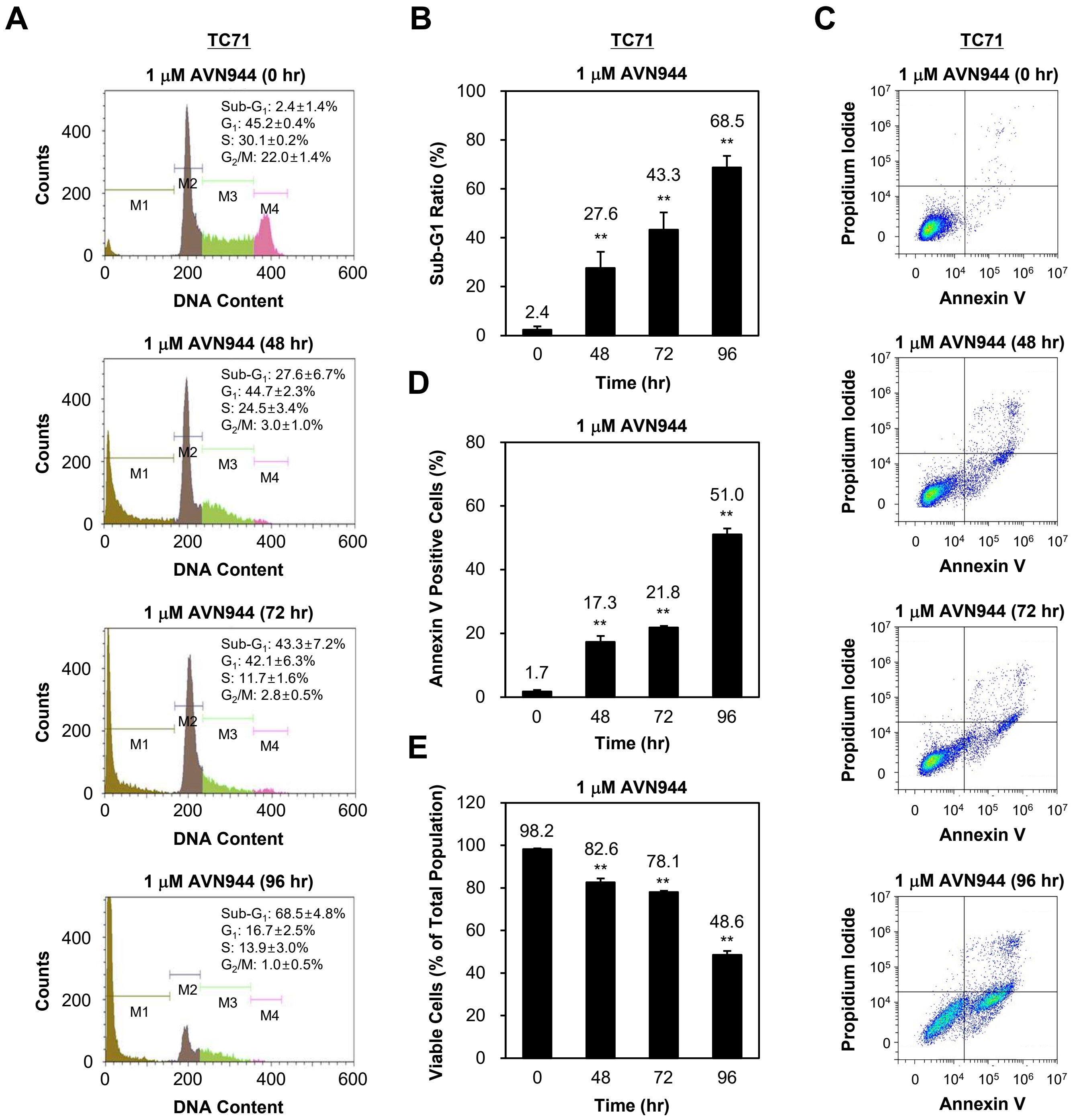

Supplementary Figure S8

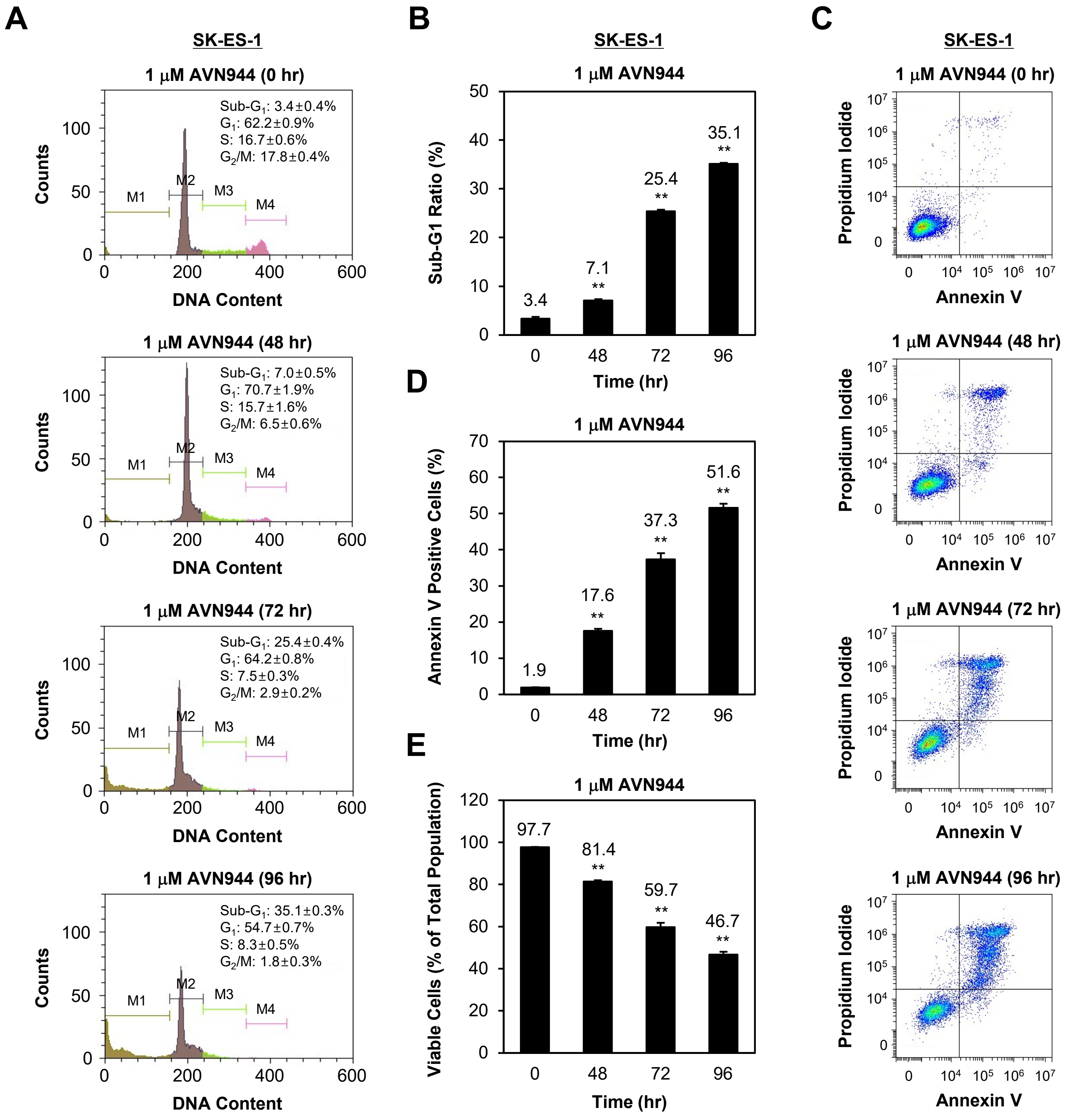

Supplementary Figure S9

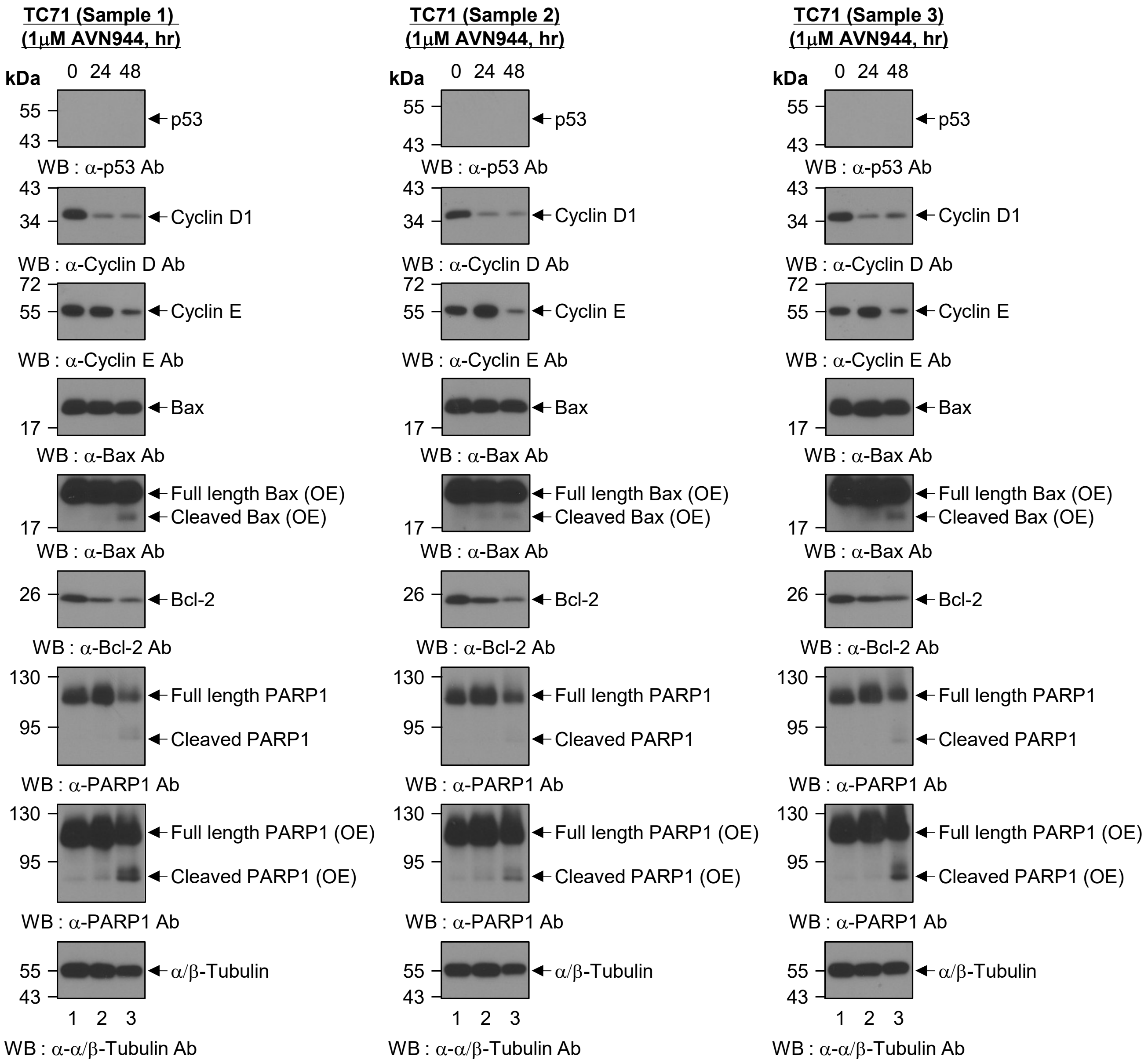

Supplementary Figure S10

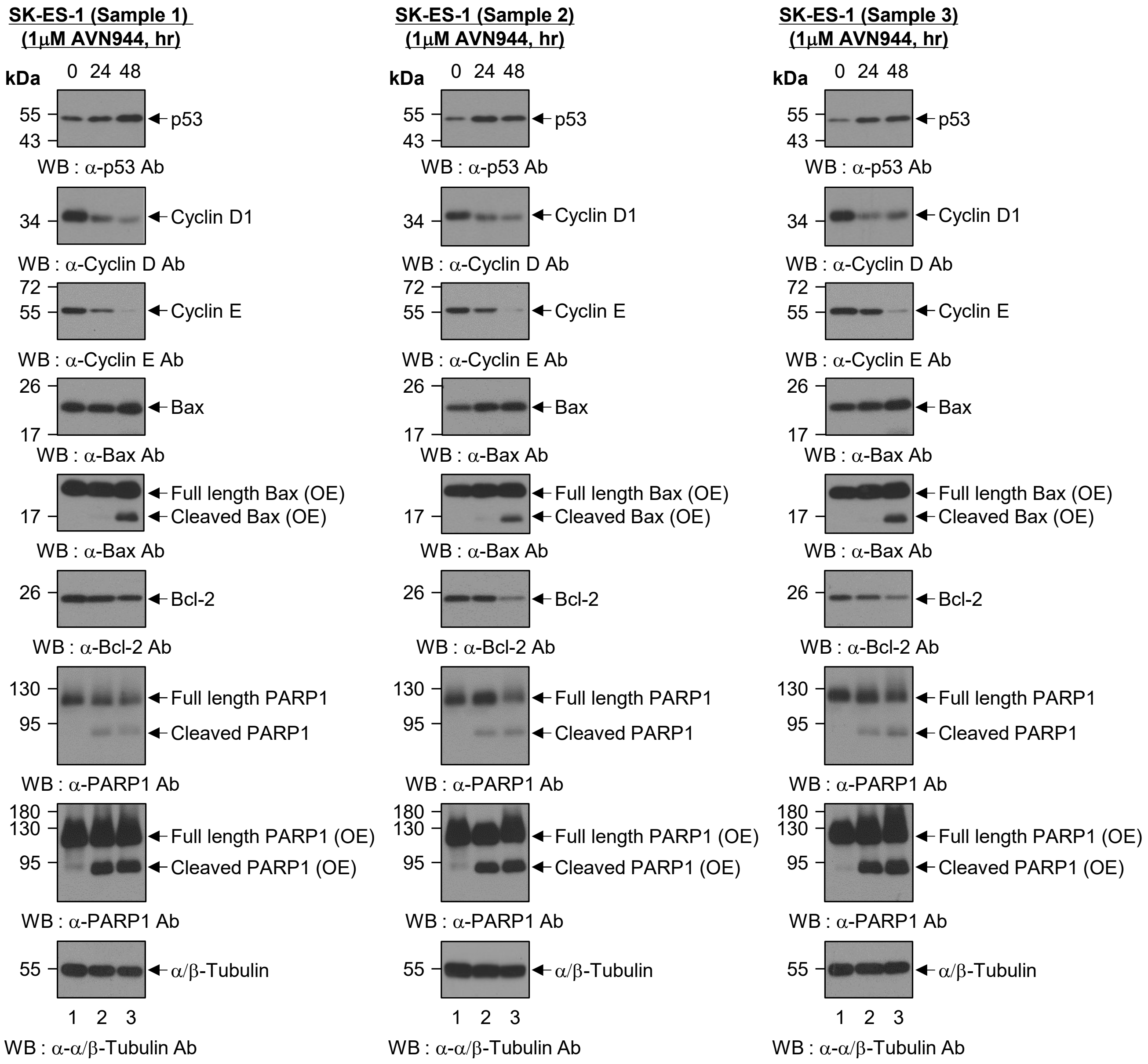

Supplementary Figure S11

Lim et al.

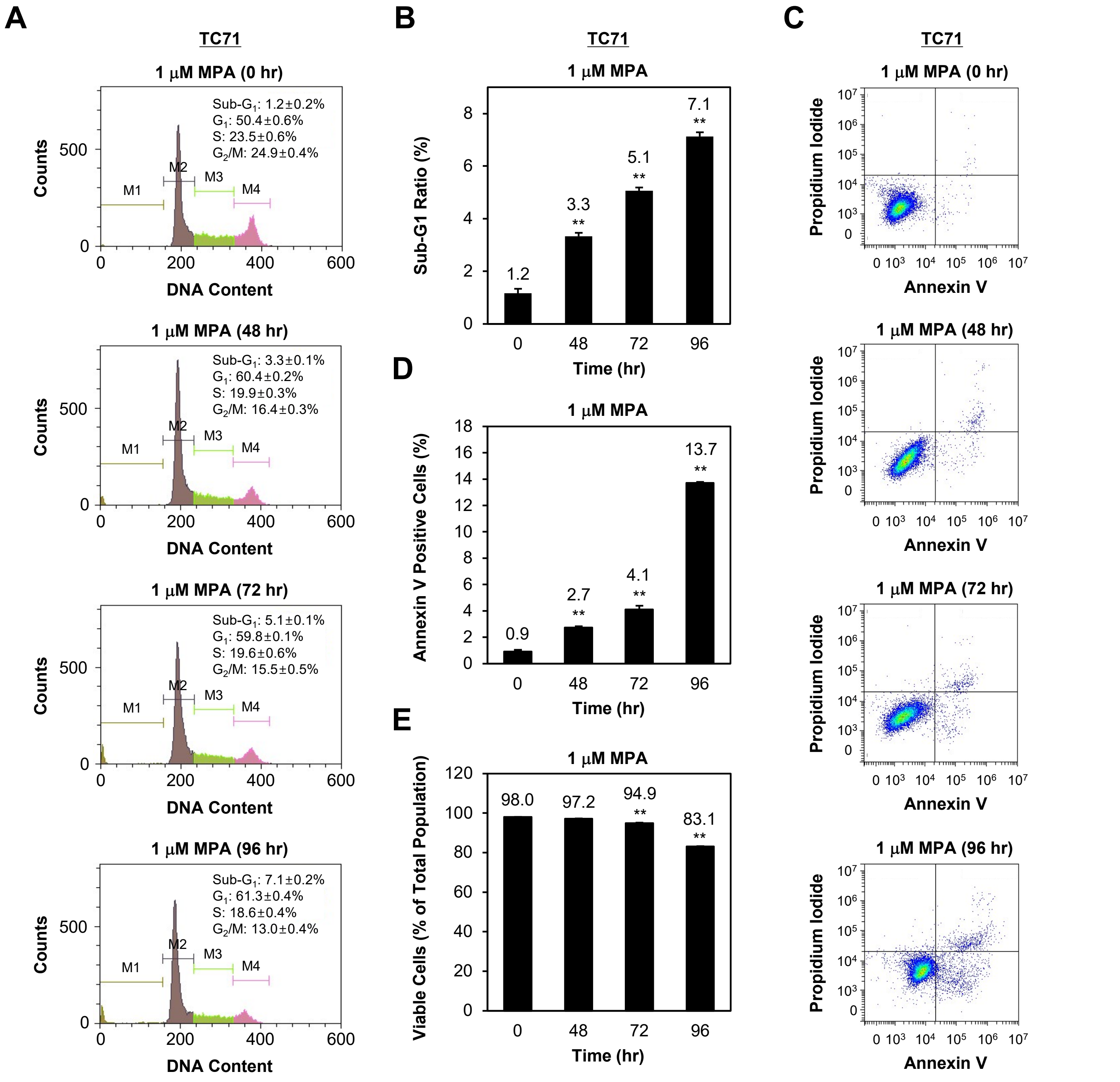

Supplementary Figure S12

Lim et al.

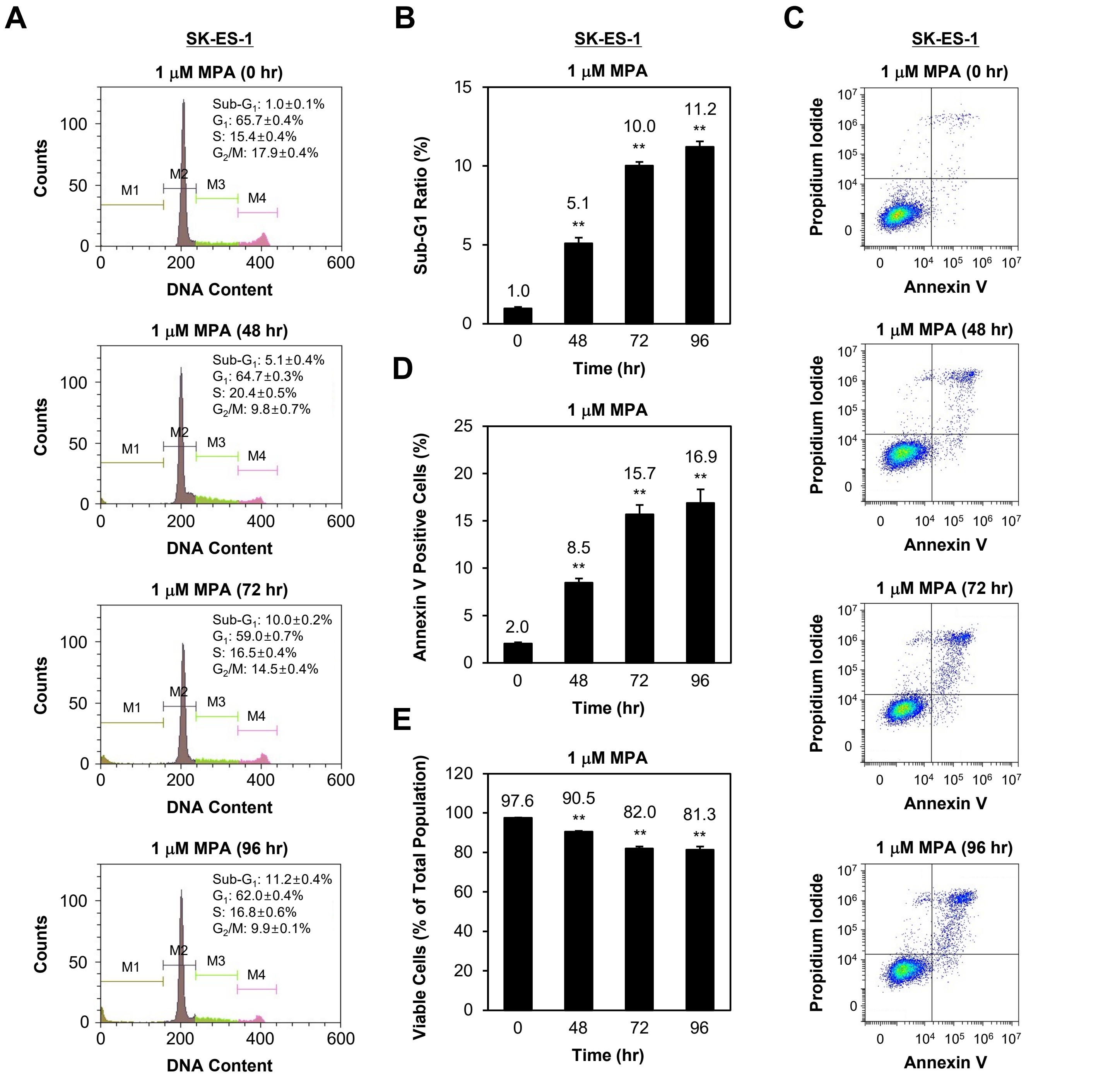

Supplement: Supplementary file 1 — Supplementary materials and methods, figures. [file ijbsv22p0529s1.pdf]
